# Use of accelerometry to measure the dynamics of activity patterns of Atlantic bluefin tuna after tagging and release

**DOI:** 10.1186/s40462-025-00563-4

**Published:** 2025-06-05

**Authors:** Jessica L. Rudd, Kim Aarestrup, Ghalia Abel, Francisco Alemany, Henrik Baktoft, Francis C. T. Binney, Samantha Birch, Kim Birnie-Gauvin, Barbara A. Block, Martin A. Collins, Owen M. Exeter, Francesco Garzon, Thomas W. Horton, Alex Plaster, David Righton, Jeroen van der Kooij, Matthew J. Witt, Serena Wright, Lucy A. Hawkes

**Affiliations:** 1 Hatherly Laboratories, University of Exeter, Prince of Wales Road, Exeter, EX4 4PS UK; 2 National Institute of Aquatic Resources, Technical University of Denmark, Silkeborg, Denmark; 3 International Commission for the Conservation of Atlantic Tunas, GBYP, Madrid, Spain; 4 Government of Jersey Marine Resources, Natural Environment, Howard Davis Farm, Trinity, Jersey; 5 Centre for Environment, Fisheries and Aquaculture Science, Pakefield Road, Lowestoft, NR33 0HT UK; 6 Department of Oceans, Stanford University, Hopkins Marine Station, Pacific Grove, CA USA; 7 British Antarctic Survey, NERC, High Cross, Cambridge, CB3 0ET UK; 8 University of Exeter, Penryn Campus, Penryn, Cornwall, TR10 9FE UK; 9 School of Environmental Sciences, University of East Anglia, Norwich, NR4 7TJ UK

**Keywords:** Biologging, Post-release behaviour, Accelerometry, Recovery period

## Abstract

Research on the direct effects of capture and tagging on post-release behaviour is typically limited to short-term deployments. To investigate the initial and longer-term behavioural responses to capture and tagging, we deployed eight Cefas G7 tags (1Hz depth and temperature, and 20 Hz triaxial acceleration) for 21–94 hours and 12 Wildlife Computers MiniPATs (depth, temperature, light and triaxial acceleration, each at 0.2 Hz) for 110–366 days on Atlantic bluefin tuna (ABT) in the English Channel. Post-release, ABT exhibited a strong, highly active initial swimming response, consistent with patterns reported in previous bluefin tuna, billfish and elasmobranch tracking studies. Accelerometry tags revealed that activity (VeDBA g), tailbeat amplitude (g) and dominant stroke frequency (Hz) were greater (2.4, 3.2 and 1.4 times respectively) within the first hour post-release than the subsequent 24 hours, stabilising at lower levels within 5–9 hours. However, lower resolution accelerometry data (0.2 Hz), obtained from longer periods from MiniPATs, revealed that fish then maintained this reduced activity for 11 ± 7.9 days (mean ± 1 SD; range: 2–26 days), during which they displayed disrupted diel patterns of activity and allocated on average 5 minutes of each day to burst energy events, compared to 14 minutes (max 74 minutes) during “recovered” periods. Subsequently, their activity levels increased again and were characterised by higher magnitude acceleration events (which may constitute feeding events) and became more active during the day than at night. Year-long deployments revealed that consistent diel vertical migration, diurnal patterns of activity, and increased time allocation to fast starts are normal for ABT off the British Isles in summer months, and their absence at the start of data collection may be related to the effect of capture and tagging, which may be longer lasting, and more complex than previously appreciated.

**Supplementary Information:**

The online version contains supplementary material available at 10.1186/s40462-025-00563-4.

## Background

Biologging of bony fish and elasmobranchs has contributed to stock and mortality assessments [1–3], welfare management in recreational fisheries and aquaculture [4, 5], protected area designation [6], invasive species control [7] and fisheries management [8, 9]. Tracking of wild fish often requires capture, tagging and handling of animals prior to release, which may induce behavioural changes [10, 11], physiological stress [12–17], or even mortality [18, 19]. Hence, addressing and mitigating the adverse effects of capture, tagging and release is needed.

Fish in captive studies can be acclimated for a period of time following handling and/or tagging prior to study [20, 21], but this is largely unfeasible in the field, particularly for large marine species. To avoid including periods of acclimation to attached or implanted devices, studies often exclude data at the start of tracking periods for a (usually arbitrary) period, ranging from hours to weeks [22–29]. However, there is a lack of baseline understanding of when sub-lethal effects subside and when the study animal returns to “normal” [30]. This understanding might be gained using high-resolution tri-axial acceleration data [31] but has rarely been achieved owing to battery power and memory capacity limitations [32]. Consequently, studies investigating post-release behaviour using accelerometry typically last from minutes [11, 33, 34] to a few days [10, 15].

Atlantic bluefin tuna (*Thunnus thynnus*, hereafter ABT) are highly migratory pelagic predators distributed throughout the North Atlantic Ocean [8, 9, 35–37], whose population biomass is increasing [38]. Whilst electronic tagging studies have provided valuable data to support management of ABT stocks [3, 39, 40], there remains only a superficial understanding of the response of ABT to capture, tagging and release over timescales longer than a few days [41–44]. Better understanding of how the capture and tagging process impacts ABT behaviour is therefore essential to maximise the value of tagging data.

Previous studies on ABT support that post-release, capture and tagging has an immediate effect on swimming behaviour, with tailbeat frequencies that were 50% higher in the first hour post-tagging and release compared to the subsequent 24 h [44] and remained high for up to 6 h [45]. Iosilevskii et al. [16] and Gleiss et al. [45] reported swimming speeds that were twice and 1.7 times higher, respectively, in the first six hours than in the hours afterwards. However, although ABT recover well from capture and tagging [3, 8, 41, 46], as evidenced by observations of important behaviours (e.g. spawning) from tagged ABT [39, 47, 48], it is unclear if the response to catch-and-release persists over a longer period (e.g. days to weeks) rather than the short periods suggested to date. This may be particularly important if ABT are caught and tagged when they are about to perform ecologically significant behaviours such as spawning, which may be disrupted as a result of the capture and tagging process. Minimising the post-release response is paramount for their welfare [49].

In the present study, we make use of long-term deployments and retrievals of archival pop-up tags to make observations on the potential long-term effects of capture and tagging on ABT tracked off the British Isles.

## Methods

### Tag deployments

Between October 2018 and November 2021, 81 ABT (mean curved fork length: 198 ± 22 cm, range: 153–242 cm) were captured off the southwest coast of England, Wales and the Channel Island of Jersey, by professional rod and reel anglers, using trolled lures attached to spreader bars and 50 or 80 lb class reels. Fishing metrics were recorded including gear type (line test, hook type), fight time (duration from initial hooking to the fish being brought alongside the boat), handling time (time from which fish were boarded until release back to the water), and water depth at release. Captured tuna were assessed for their fitness for tagging and again for their fitness for release back to the wild (Supplementary Fig. 1). On-deck, their gills were irrigated with saltwater while the tagging procedure took place (for detailed handling methods, see [37]) and they were then returned to the water and towed using a ‘boga grip’ (Pratiko, Italy) or lip hook at the side or rear of the vessel < 5 knots to aid reoxygenation. Fight times ranged from 10 to 34 min, handling times ranged from 1.3 to 3.5 min (mean 2.6 ± 0.5 min), and all ABT were towed for 4.3 ± 1.4 min (range 1.3–7.3 min) before release (Table 1). Eleven ABT were tagged with packages that comprised a Cefas G7 pDST accelerometer tag (Cefas Technology Limited CTL, UK), coupled with a mrPAT or mrSPOT tag (model 375, Wildlife Computers, WA, USA) to facilitate recovery of the tag following its release from the ABT. These packages were attached front and aft using cable ties to a saddle of two galvanic time releases, which corroded over a period of 2–4 days, eventually releasing the tag package. G7 tags were programmed to collect temperature and depth at 1 Hz frequency, and tri-axial acceleration at 20 Hz (*n* = 9 fish) or 30 Hz (*n* = 2 fish). Data gathered at 30 Hz were decimated to 20 Hz through linear interpolation using the R package “dplyr” so that all G7 tags could be analysed at the same frequency. A further 70 fish (mean curved fork length: 197 ± 23 cm, range: 153–242 cm) were tagged with Wildlife Computers MiniPAT tags, attached by a monofilament tether to a titanium dart inserted intramuscularly near the second dorsal fin. The tag was held in place with an additional loop that was also attached by an intramuscular titanium dart [37, 50]. MiniPAT tags recorded depth, temperature, tri-axial acceleration and light level at 0.2 Hz (1 data point every 5 s) or 0.067 Hz (one data point every 15 s) for a year. A total of 27 MiniPAT tags and 8 G7 tags were retrieved after detachment from the ABT. By down-sampling data from the high-resolution tags to various frequencies (ranging from 20 Hz to 0.05 Hz), tailbeat signal from the lateral acceleration was lost below 5 Hz and bursts in acceleration could only be detected above 0.2 Hz. Thus, the fifteen MiniPAT tags recording at 0.067 Hz were excluded from the present study as the resolution was deemed too low to retrieve post-release behaviour. A total of 20 recovered tags (*n* = 8 G7 tags and *n* = 12 MiniPAT tags) were then included in the present study (Table 1). The Wildlife Computers Global Position Estimator 3 (GPE3) light geolocation model (https://static.wildlifecomputers.com/Location-Processing-UserGuide.pdf) was used to generate 0.25 × 0.25° location likelihood rasters for the MiniPAT datasets, where cells with < 1% likelihood were omitted from further analysis. Mean bathymetry was extracted from General Bathymetric Chart of the Ocean (GEBCO, gebco.net; resolution 0.004°) using the R package “raster”. Mean bathymetry for each location was expressed as the grand mean depth of each raster cell, weighted by the likelihood values.

**Table 1:** Electronic tag deployments on Atlantic Bluefin Tuna (one fish per row) tagged in the south-west UK (2018–2021). * NR refers to no complete record of tow duration

Tuna ID	Tag type	Sampling frequency	Deployment date	Deployment duration (days)	Displacement distance (km)	Tag location to pop-up distance (km)	Curved Fork length (cm)	Estimated weight (kg)	Fight time (min)	Tow time (min)
16P1231	MiniPAT	0.2 Hz	23 Aug 2019	265	11,417	1208	212	148	15	03:22
16P2365	MiniPAT	0.2 Hz	25 Aug 2019	121	3649	682	199	116	11	04:11
17P1004	MiniPAT	0.2 Hz	03 Sept 2019	365	16,224	135	203	134	12	04:59
18P0812	MiniPAT	0.2 Hz	02 Nov 2018	278	13,432	288	175	81	18	NR
18P0837	MiniPAT	0.2 Hz	02 Sept 2019	365	15,745	794	181	90	15	03:33
18P0932	MiniPAT	0.2 Hz	23 Oct 2019	337	14,134	35	238	211	34	03:11
19P0137	MiniPAT	0.2 Hz	18 Nov 2019	314	8964	77	166	80	NA	NR
19P0206	MiniPAT	0.2 Hz	02 Oct 2019	362	19,599	7	221	141	28	05:40
20P0084	MiniPAT	0.2 Hz	09 Sept 2020	362	14,145	44	229	155	12.67	04:59
20P1136	MiniPAT	0.2 Hz	12 Sept 2020	365	15,733	193	194	107	12.68	05:03
20P1137	MiniPAT	0.2 Hz	16 Sept 2020	359	14,520	197	201	129	18	07:16
21P0468	MiniPAT	0.2 Hz	04 Sept 2021	110	2016	224	153	51	10.88	NR
A15884	G7 pDST	30 Hz	08 Sept 2019	3.93		380	193	113	10	NR
A17240	G7 pDST	20 Hz	09 Sept 2020	1.85		175	196	98	11.12	03:08
A17247	G7 pDST	20 Hz	09 Sept 2020	0.89		172	205	127	15	04:46
A17248	G7 pDST	30 Hz	21 Oct 2019	2.15		177	212	152	30	NR
A17890	G7 pDST	20 Hz	11 Sept 2020	3.73		172	212	124	10	01:16
A17891	G7 pDST	20 Hz	15 Sept 2020	3.64		173	204	131	10	03:00
A17893	G7 pDST	20 Hz	13 Sept 2020	3		113	207	149	30	05:52
A17939	G7 pDST	20 Hz	15 Nov 2021	3.68		158	185	98	10	03:54

### Data processing

To account for differing tag attachment orientation on each tuna, accelerometry data were calibrated following rotations of known angles using the R package “tagtools”, once data were processed to 20 Hz for G7 tags. This enables each tag’s frame of reference to be aligned with the tuna’s axes and be comparable between individuals. The accelerometry data from the MiniPAT (collected a 0.2 Hz) were processed in the same way. Depth was temperature corrected using the R package “tagtools”. “Activity” was defined using accelerometery data, which comprises two components, (i) low-frequency static acceleration and (ii) high-frequency dynamic acceleration. The static component relates to the inclination of the tag with respect to the earth’s gravitational field (which is analogous to the ABT’s body posture) and was obtained by individually smoothing each of the three acceleration channels with a running mean of two seconds for the G7 tags (following [51]). Dynamic acceleration relating to the tuna’s movement [52] was obtained by subtracting the static acceleration from the raw acceleration in all three axes and then expressed as Vectorial Dynamic Body Acceleration (VeDBA; [53]). A spectrogram of the lateral acceleration was generated in Ethographer ver. 2.04 [54] in Igor Pro (Igor Pro 8, WaveMetrics Inc., Lake Oswego, USA), calculated by continuous wavelet transformation using the Marlet wavelet function with a minimum cycle of 0.125 s and maximum cycle of 2 s for each fish [54]. Tailbeat amplitude (TBA; g) was calculated for each 1 s interval using the Peak Tracer function in Igor Pro. Dominant stroke frequency (DSF) was calculated using the R package “tagtools”, with tailbeat period (TBP) calculated as the inverse of DSF, in seconds. TBA, TBP and DSF for each ABT were divided by the curved fork length to correct for size. Since ABT tailbeat signal is lost below 5 Hz sampling frequency, TBA, TBP and DSF metrics were only extracted for the G7 tags. To investigate whether the activity of G7-tagged ABT differed from those tagged with MiniPATs, G7 data were down-sampled to 0.2 Hz to match the MiniPAT tags and expressed using VeDBA calculated over a 10 s smoothing window (Supplementary Fig. 2). Due to differences in attachment styles between tag types, fine scale movement of the tag (“wobble”) differed slightly between tag types (see discussion below).

### Immediate response post-release

For G7 tags, a mean value was calculated for VeDBA, TBA, DSF and TBP for each hour, per fish, for the first 24 h of deployment, and metrics were deemed to have stabilised once mean values had plateaued (right-sided asymptote). The overall plateau time, representing the mean timing at which swimming metrics plateaued, was calculated across all fish. Mean hourly VeDBA was also calculated for MiniPAT tags and for the down-sampled G7 accelerometry data for each hour of deployment. Trends in mean hourly activity for the first 24 h post-release (considered ‘immediately post-release’) were visualised using the “geom_smooth” function in R using the formula y ~ s(x) from the family “loess”. Other large billfish and tuna species have previously been observed making an initial, sudden near vertical dive following release, which is thought to be linked to a physiological stress response, and/or perhaps thermoregulatory behaviour. They often remained at depth for an atypically long period before returning to shallower waters [55–57]. In the current study, the duration of the first dive (defined as > 10 m and lasting at least 20 s, before returning to < 10 m, following [57]) was plotted against fight time. Depth data from G7 tags were down-sampled to match the sampling frequency of the MiniPAT tags. Depth data for both tag types were then smoothed to a 10 s running mean.

### Longer term patterns of behavior

To determine whether activity and depth patterns of each ABT for the first seven days following capture and tagging differed from the overall behavioural patterns observed in the first month post-release, a resampling test using similarity values was performed following [19] on the 12 MiniPAT tags. Pair-wise correlation coefficients of mean hourly VeDBA and depth values for MiniPAT data were calculated daily, for the first and last 30 days post-release separately (i.e. day 1 against day 2, day 1 against day 3, ... day 30 against day 1 etc.) using the function “ccf” in R with zero lags, with activity and depth analysed separately. Cross-correlation values ranged between − 1 and 1 (perfect negative and positive correlation, respectively), while values of 0 indicated no association. Median correlation values (from here on ‘daily similarity values’) were then calculated for each day. Days with dissimilar patterns of behaviour would be expected to have values lower than 0. Days were then ranked from 1 to 30 based on daily similarity values, from lowest (day most dissimilar to the overall pattern) to highest (day most similar to the overall pattern). The rank of daily similarity values for each fish was shuffled 10,000 times without replacement using the R package “resample” and the first seven values from each reiteration sampled to represent days 1–7 post-release. Values falling below the 5th percentile of the distribution of resampled values for each day were considered significantly different to the overall behavioural pattern. Following [19], post-release behaviour in the first week following release was considered different if at least one of the first seven days’ similarity values was lower than the 5th percentile of resampled values, as well as the average rank of the first 7 days being below 15 (i.e. the lower half of correlation values) (see Tables 2 and 3).

**Table 2:** Correlation and similarity ranks of ABT diel activity patterns relative to overall post-release, for all 12 fish for the first 30 days post-release and 6 fish (with year-long deployments) for the last 30 days. * Fish ID in bold indicates potentially altered activity patterns during the first 7 days post-release

Fish ID	Correlation coefficient range	Dissimilar post-release days (≤ 5th percentile)	Average rank of daily similarity values among post-release days 1–7 (scale 1–30)
**First 30 days of deployment**			
**16P1231**	-0.35;0.39	2, 21	13
16P2365	-0.31;0.44	20, 21	17
17P1004	-0.30;0.28	13, 21	18
**18P0812**	-0.22;0.50	1, 7	9
18P0837	-0.16;0.29	12, 14	16
**18P0932**	-0.35;0.54	1, 6	9
19P0137	-0.14;0.20	20, 29	19
**19P0206**	-0.31;0.30	5, 15	9
**20P0084**	-0.33;0.55	4, 5	8
**20P1136**	-0.37;0.39	2, 4	5
**20P1137**	-0.08;0.45	3, 5	8
**2,130,468**	-0.18;0.76	3, 4	6
**Last 30 days of deployment**			
17P1004	-0.09;0.53	20, 21	16
18P0837	0.38;0.71	17, 30	19
19P0206	0.48;0.82	19, 27	14
20P0084	0.54;0.80	1, 9	11
20P1136	0.38;0.81	17, 18	20
20P1137	-0.07;0.76	18, 21	16

**Table 3:** Correlation and similarity ranks of ABT diel depth-use patterns relative to overall post-release, for all 12 fish for the first 30 days post-release and five fish (with year-long deployments) for the last 30 days

Fish ID	Correlation coefficient range	Dissimilar post-release days (≤ 5th percentile)	Average rank of daily similarity values among post-release days 1–7 (scale 1–30)
**First 30 days of deployment**			
16P1231	-0.27;0.29	25, 27	18
**16P2365**	-0.25;0.33	4, 25	8
**17P1004**	-0.27;0.21	2, 28	15
18P0812	-0.23;0.32	27, 30	14
18P0837	-0.17;0.44	4, 12	14
18P0932*	-0.12;0.48	3, 9	8
19P0137	-0.18;0.53	20, 29	21
19P0206*	-0.26;0.33	3, 5	9
20P0084	-0.26;0.11	23, 24	18
20P1136	-0.11;0.18	16, 24	18
20P1137	-0.08;0.34	15, 21	17
2,130,468	-0.31;0.43	20, 21	23
**Last 30 days of deployment**			
18P0837	-0.17;0.60	12, 26	11
19P0206	0.27;0.84	1, 2	13
20P0084	0.29;0.72	9, 10	12
20P1136	0.35;0.54	9, 10	14
20P1137	0.18;0.26	4, 11	14

Fish ID in bold indicates potentially altered depth patterns during the first 7 days post-release

* Fish that also had altered patterns of activity

Since fish exhibited inconsistent behavioural patterns, as indicated by low daily similarity values and a lack of clear diel behaviour (see “Overall diel patterns of activity and depth” methods below) for periods extending beyond the first week, a criterion was established to determine when they resumed a consistent pattern of diel behaviour. Return of diel behaviour was defined as occurring when fish displayed at least three days of diurnal behaviour within a seven-day window (e.g. vertical red line in Fig. 2). Owing to small sample sizes and multiple possible interacting variables, ABT length, weight, fight time, total handling time, ambient temperature and lunar phase were tested independently for influence on the timing of the return of cyclical pattern of activity, using linear regressions.

Finally, the 11th month of tracking data (last 30 days of deployment for fish with over 330 days tracking days, *n* = 6 fish) was used as a control to compare activity and depth patterns with the first 30 days post-release. By this point, ABT had returned to locations similar to where they were initially captured and were experiencing conditions and bathymetry comparable to those prior to tagging. While the sublethal effects of capture and tagging were expected to have subsided within a month, ABT activity and depth use are known to vary with migration phases [58], so behavioural patterns observed outside of their English Channel feeding grounds may not reflect how the ABT would typically be behaving had capture and tagging not occurred. We therefore excluded any comparisons of months 2–10 post-release due to the ABT likely being in different places or at different times to when there were initially tagged.

### Impact of capture, tagging and release on the spatiotemporal variation in “fast start” events

‘Fast starts’ are sudden, brief, acceleration bursts, potentially associated with predator-prey encounters and/or other feeding activities [59]; though without simultaneous video validation [60] or archival tags measuring the heat increment of feeding [61], foraging behaviour cannot be confirmed. Fast starts were determined in the present study following methods in [62] as events where VeDBA values were above the 99th percentile of the entire deployment period for MiniPAT tags (Supplementary Fig. 3). The time allocated to fast start events per week during the first and last 30 days post-release was then compared.

### Overall diel patterns of activity and depth

Sunrise and sunset times derived from light levels (via GPE3 outputs) were used to partition periods of day and night. For each MiniPAT tagged ABT (*n* = 12), a mean VeDBA and mean depth value was calculated for each day and night periods, and diel patterns of activity tested using Welch t-tests, or Wilcoxon rank sum tests if data were not normally distributed. Activity was classified as either diurnal, nocturnal, or neither, and average daily depth values were compared for day and night-time periods using Welch t-tests or Wilcoxon rank sum tests. Differences in VeDBA and mean depth were compared for the first and last 30 days of deployment for the six ABT that had year-long MiniPAT deployments. To test whether the lunar cycle influenced the overall depth use and activity in the first and last 30 days of deployment, moon phase was extracted using dates with the R package “lunar”. Then, mean depth and activity were compared between moon phases using Kruskal Wallis rank-sum tests. The effect of bathymetry on depth use, and the timing of when diel depth behaviour returned, was also examined.

### Movement

Minimum straight-line distance between daily locations from MiniPAT data were calculated using the “oce” R package, and a straightness index (SI) was calculated as the minimum horizontal distance between release and pop-off locations, divided by the cumulative distance travelled by the ABT between these points [63]. SIs were calculated for the first and last 30 days of deployment, as well as weekly for the first and last four weeks of the deployment, and compared using Welch t-tests. ABT daily locations were classified into two latent categorical states: (1) directed movement and (2) localised movement, using Hidden Markov Models (HMM) in the R package “MoveHMM” R [64]. Step lengths were modelled using a Gamma distribution, and turning angles were modelled using a Von Mises distribution. Directed swimming was associated with larger travel distances and directional persistence, where turning angles were close to 0, while localised movement was assumed to be associated with short travelling distances and high turning angles. Models were checked using the pseudo-residuals [64]. To test whether patterns of depth use were influenced by movement states, mean daytime and nighttime depths were compared for periods of directed and localised swimming for both the first 30 days and the last 30 days of deployment separately. Generalised linear mixed models (GLMM) were fitted to the data with mean depth as the response variable. Daytime or nighttime, and behavioural state (directed and localised movement) were included as fixed effects, while variation between fish was accounted for by specifying tag ID as a random effect. GLMMs were fitted with log-transformed data using the “lme4” package in R. Models were checked by visually inspecting standardised residuals using the “Performance” package.

## Results

### Behaviour immediately following release

**Fig. 1:**
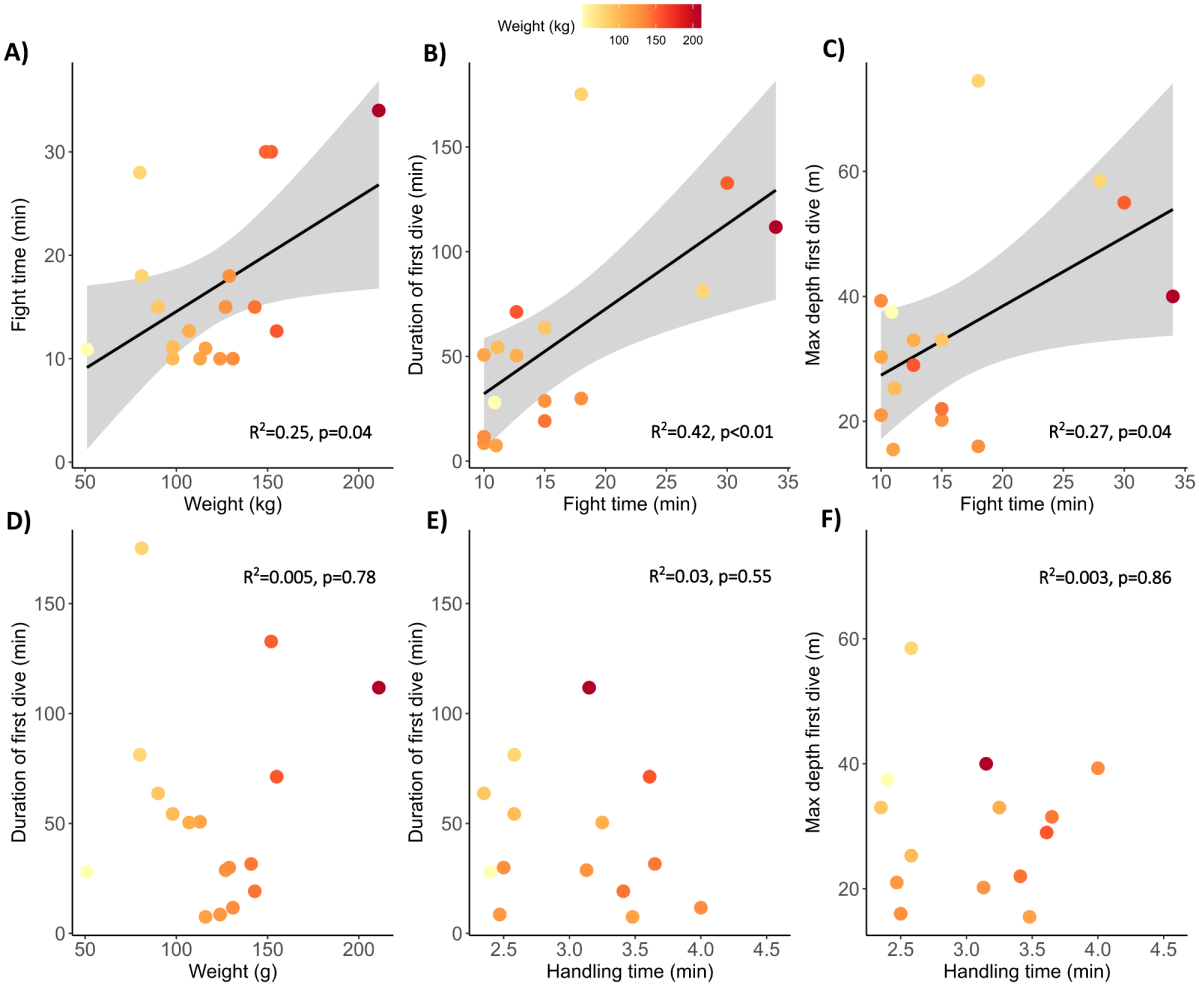
Effects of capture and tagging on duration and depth of Atlantic bluefin tuna first dive. Scatterplots showing the relationship between (first row) fight time and (**A**) estimated fish weight, (**B**) the duration of the tuna’s first-dive post-release, and (**C**) the maximum depth of the first dive (defined as the first dive below 10 m lasting at least 20 s). (Second row) the relationship between the duration of first dive following release (y-axis) and (**D**) total handling time (fight time and time spent on deck), (**E**) duration of the first dive and handling time (time spent on deck), and (**F**) maximum depth of he first dive (m) and handling time. Each circle represents a single fish colour-coded by its weight (kg, where redder colours indicate heavier fish). Grey polygons indicate 95% confidence intervals of linear relationship (black line)

Fight time increased significantly for heavier ABT (linear regression, y = 0.11x + 3.52; R^2^ = 0.25, *p* = 0.04, Fig. 1A). Following release, most ABT made an initial dive to 34 ± 15.9 m (mean ± SD, range 16–75 m, mean seafloor depth at site of release 69 ± 13.5 m, range: 43–107 m) for between 8 and 175 min (mean 51.7 ± 46.5 min). While estimated weight had no influence on dive duration (linear regression: y = 0.09x + 45.04; R^2^ = 0.005, *p* = 0.78, Fig. 1D), fight time significantly increased dive duration (linear regression: y = 4.05–8.35; R^2^ = 0.42, *p* < 0.01; Fig. 1B), with each minute of fighting resulting in 4.05 ± 1.27 min longer dives. Handling time at the vessel did not have a significant effect on dive duration (linear regression: y=-9.73x + 72.28; R^2^=-0.03, *p* = 0.55, Fig. 1E) nor the maximum depth of the dive (linear regression: y=-1.10x + 33.48; R^2^ = 0.003, *p* = 0.86, Fig. 1F). The maximum depth reached by ABT in the initial dive also increased significantly with fight time (linear regression, R^2^ = 0.27, *p* = 0.04, depth = 1.11 fight time + 16.32; Fig. 1C).

**Fig. 2:**
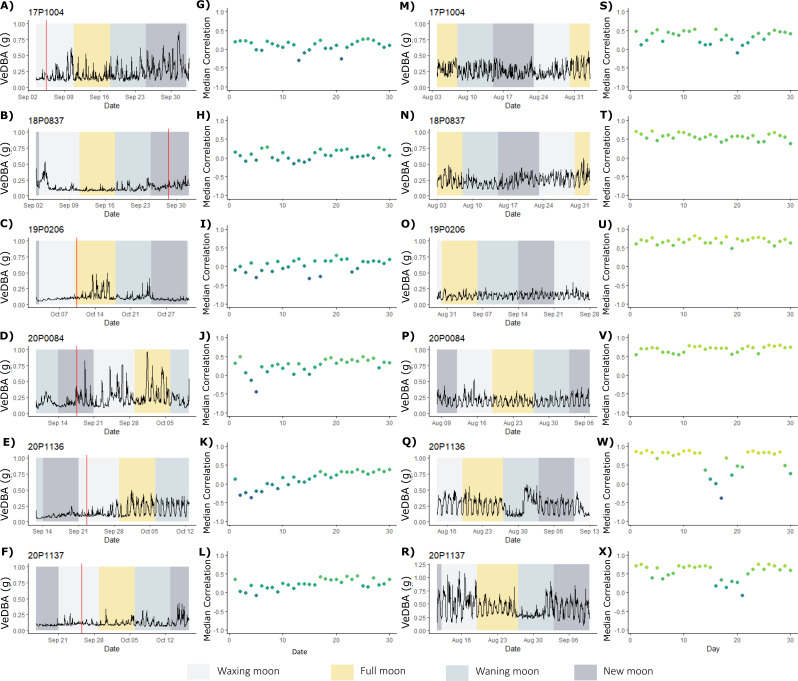
ABT activity patterns for the first and last 30 days of deployment. Mean hourly activity (VeDBA) pattern (first and third columns) of six fish (one fish per row) for the first 30 days (**A**-**F**) and last 30 days (**M**-**R**) of deployment. Shaded backgrounds correspond to the lunar phase (see legend, bottom). Red vertical lines show the day on which regular DVM was exhibited (figures lacking red line did not exhibit disrupted depth patterns in the first week post-release). Daily similarity values (second and fourth columns) of each day post-release, relative to the whole deployment period for the first 30 days (**G**-**L**) and last 30 days (**S**-**X**), coloured by correlation values, where days more similar to the overall activity pattern are shown in lime green, and more dissimilar days in blue

**Fig. 3:**
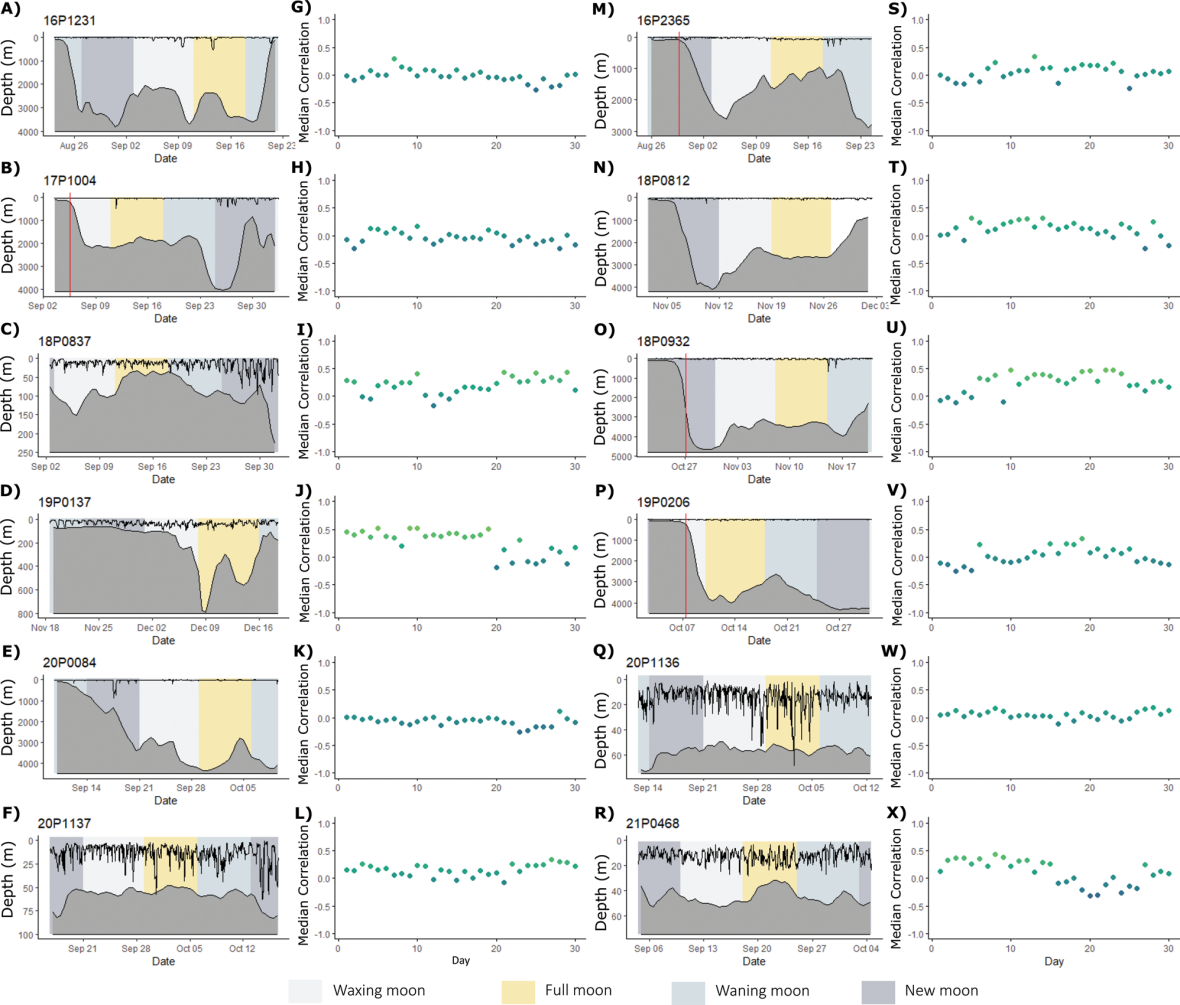
Depth use for the first 30 days of deployment for all ABT. Post-release depth profiles (first and third columns) of the twelve MiniPAT tagged fish (**A**-**F**) and (**M**-**R**), with underlying bathymetry shown as a grey polygon. Shaded backgrounds correspond to the lunar phase (legend, bottom). Red vertical lines (for four fish) show the day on which depth use patterns become more similar to the overall tracking period. Plots lacking red lines (eight fish) did not exhibit dissimilar depth patterns in the first week post-release based on daily similarity values and resampling technique described in the Methods. Daily similarity values (second and fourth columns) of each day post-release, relative to the whole deployment period (see Methods) for the first 30 days (**G**-**L** and **S**-**X**), coloured by correlation values, where days more similar to the overall activity pattern are shown in lime green, and more dissimilar days in blue

**Fig. 4:**
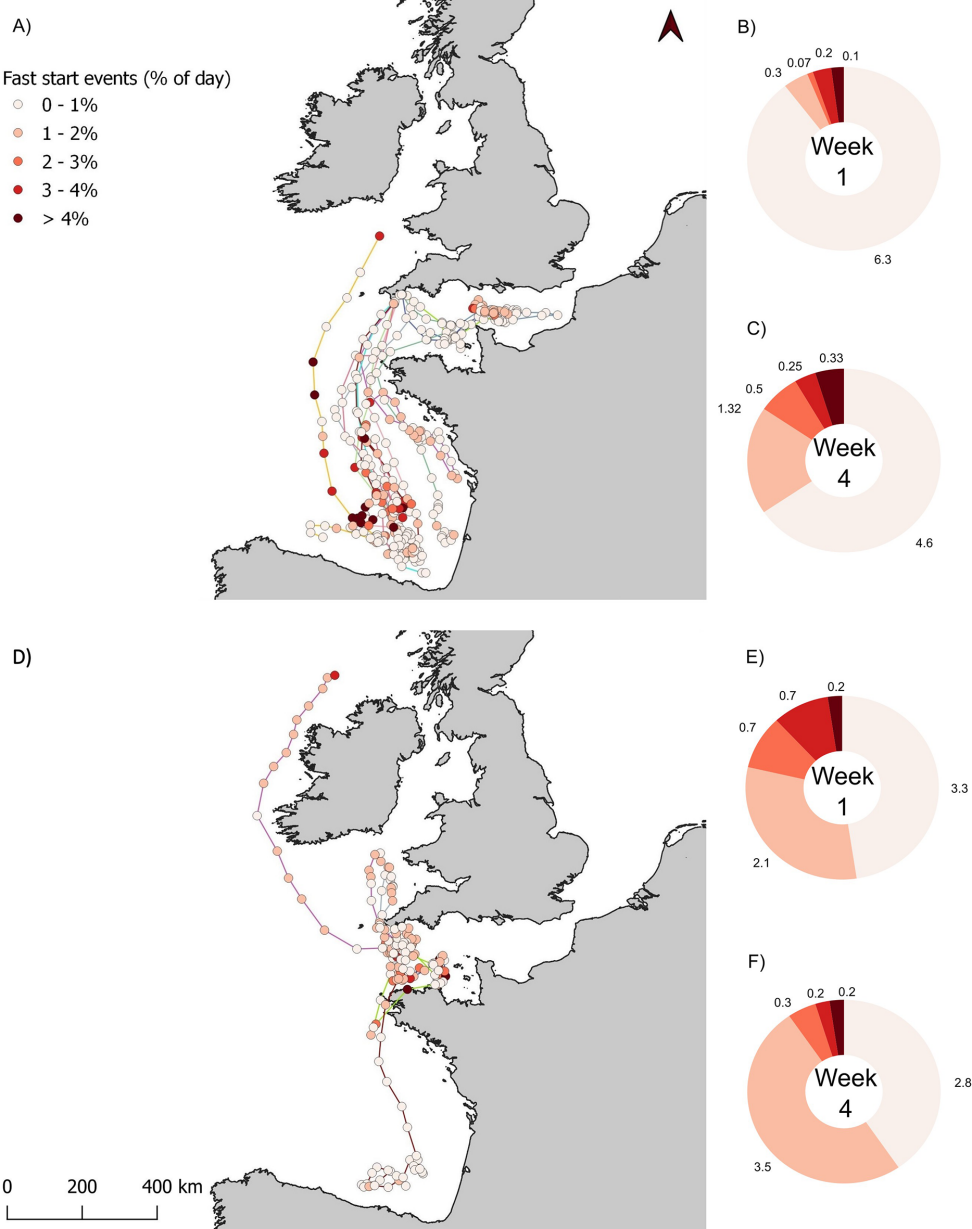
Time allocation to fast start events for the first and last 30 days of deployment. Maps of ABT daily locations for the (**A**) first (*n* = 12 ABT), and (**D**) last 30 days of the deployment (*n* = 6 ABT that collected data for 1 year). (**D**) One ABT is recorded migrating from the Bay of Biscay to the Channel, and another recorded migrating from the Channel to the west coast of Ireland where its tag popped off. Daily locations are coloured by the proportion of each day spent conducting fast start events (the percent of time that VeDBA was above the 99th percentile of values across the full tracking deployment per ABT). Ring plots represent the average number of days per week that ABT allocated to fast start events, for the first 7 days (**B** & **E**) and last 7 days (**C** & **F**) of the first and last 30-day periods respectively

ABT displayed an initial flight response following release. The greatest distance covered by the ABT occurred on the first day post-release (mean: 87.4 km ± 44.04 km in a day), followed by strongly directed swimming identified by Hidden Markov Model (HMM, slope directed movement to localised movement: -1.83 (CI: -2.83; -1.62), slope localised movement to directed movement: -3.06 (CI: -3.29; -2.05)) lasting on average of 8.3 ± 3.9 days, with a straightness index of 0.83 ± 0.08. ABT travelled on average 73 ± 14.7 km.day^− 1^ during periods of directed movement compared to 22.8 ± 22.8 km.day^− 1^ during periods of localised movement. Eight fish made directed movements for up to 14 days (straightness index: 0.66 ± 0.28), where they travelled on average 552 km (± 26 km range: 187–813 km) away from their tagging location regardless of tagging year or month (late August to mid-November) (Figs. 2, 3 and 4). Their movements then became significantly more tortuous, with a lower mean straightness index (SI_week3_= 0.48, Welch t-test, t = 4.95, *p* < 0.001, and SI_week4_=0.40, t = 5.49, *p* < 0.001 respectively; Figs. 2 and 4).

### Behaviour in the 24 h following release

**Fig. 5:**
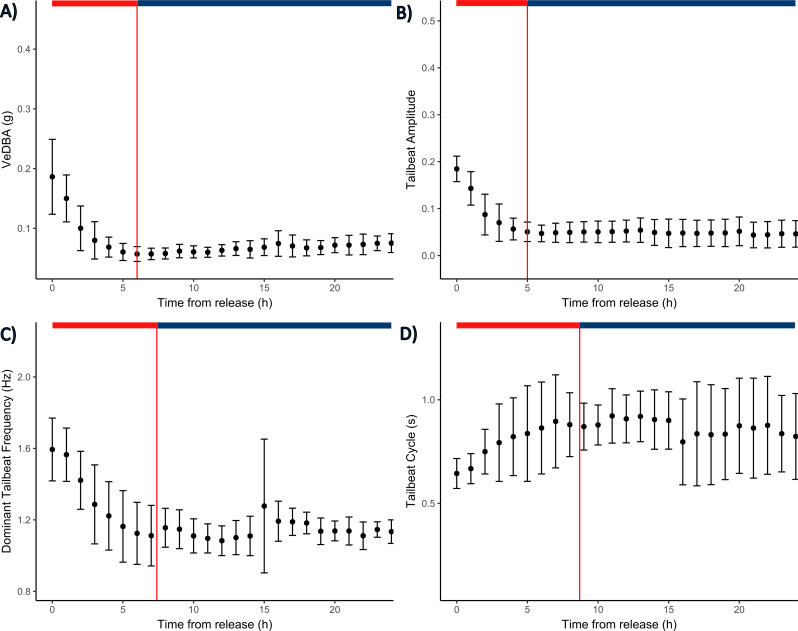
Swimming behaviour of Atlantic bluefin tuna in the 24 h following tagging. Shown are hourly average (**A**) VeDBA, (**B**) tailbeat amplitude, (**C**) dominant stroke frequency, and (**D**) tailbeat cycle (*n* = 8 G7 tagged fish). Black dots represent hourly means with bars as standard deviation. The immediate response to tagging is highlighted by the red horizontal segment after which behaviours begin to plateau, denoted by the red vertical line. The stabilisation period is highlighted by the blue segment

**Fig. 6:**
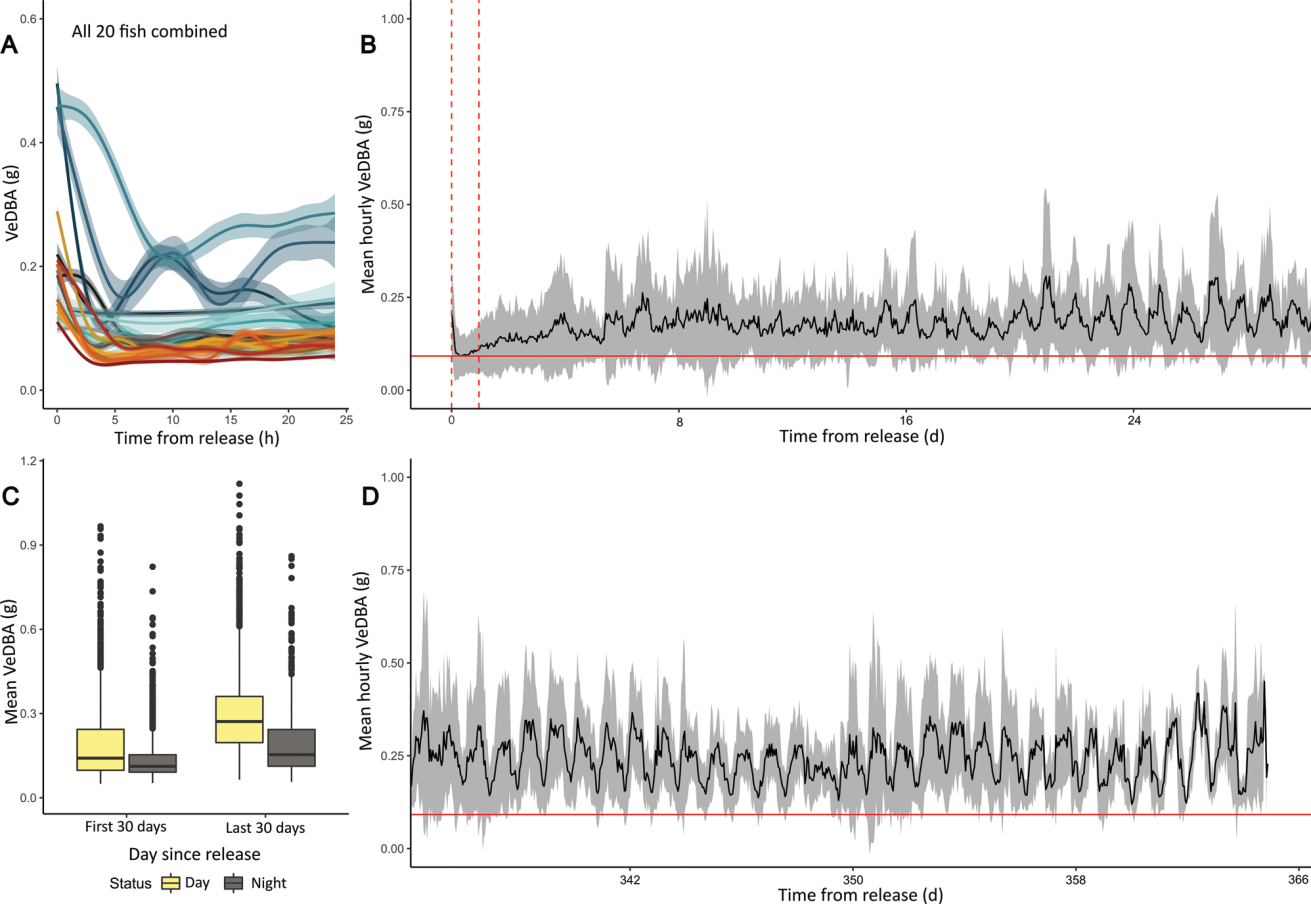
Activity of bluefin tuna post-release across different timescale. (**A**) mean hourly VeDBA (g) for the first 24 h post-release for all 20 ABT (lines show GAM models, each fish shown in a different colour, where lines in red to orange represent G7 tagged fish, while blue lines show MiniPAT tagged ABT). Shading corresponds to the 95% confidence interval for the fitted curves. (**B**) Mean hourly VeDBA for the first 30 days following release for all fish combined (grey shading shows s.d.). Time of release is represented by 0 on the x-axis, and dashed vertical lines denote the first 24-hours of the deployment. The solid red horizontal line denotes the minimum hourly mean VeDBA value for the whole tracking period, which is reached 8 h post-release, with all subsequent activity higher than this. (**C**) Boxplots showing diel activity patterns (shown as average daily VeDBA for day and night periods) between the first and last 30-day periods. (**D**) Mean hourly VeDBA for the last 30 days of deployment for the six fish with year-long deployments. The red horizontal line denotes the minimum hourly mean VeDBA value as in (B), note mean VeDBA is never lower than this line

ABT tagged with G7 tags were 2.4 ± 0.53 times more active in the first hour post-release (VeDBA: 0.18 ± 0.06 g, range: 0.11–0.30 g) than the subsequent 24 h (VeDBA: 0.07 ± 0.01 g; Fig. 5A). Tailbeat amplitude was also 3.2 ± 0.83 times greater (0.17 ± 0.04 g, vs. 0.06 ± 0.02 g) and dominant stroke frequency 1.4 ± 0.15 times greater (1.61 ± 0.16 Hz, vs. 1.18 ± 0.06 Hz, Fig. 5B). While variable, within 5–9 h post-release the metrics had declined to a relatively stable value, mean hourly VeDBA for all fish combined plateaued after 6 h (± 1.7, range 4–8 h), TBA within 5 h (± 1.5, range 3–7) DSF within 7.4 h (± 2.8 range 4–12 h) and TBP within 8.4 h (± 4.2, range 4–13 h). Similar patterns of activity were recorded for ABT tagged with MiniPATs, with mean hourly VeDBA peaking in the first hour post-release for most ABT (0.23 ± 0.09 g; Fig. 6A, Supplementary Fig. 2B), though four fish had slightly elevated mean hourly VeDBA during the third hour post-release compared to the first hour (0.05 ± 0.04 g). ABT activity declined and stabilised within 6 ± 1.9 h (range 3–9 h) (Fig. 6A). While MiniPAT and G7 tagged ABT had similar curved fork length (Welch t-test t = 0.50, *p* = 0.62), mean hourly VeDBA was 1.3 times greater for MiniPAT compared to G7 tagged fish (Supplementary Fig. 2).

### 30-day behavioural response– activity levels

**Fig. 7:**
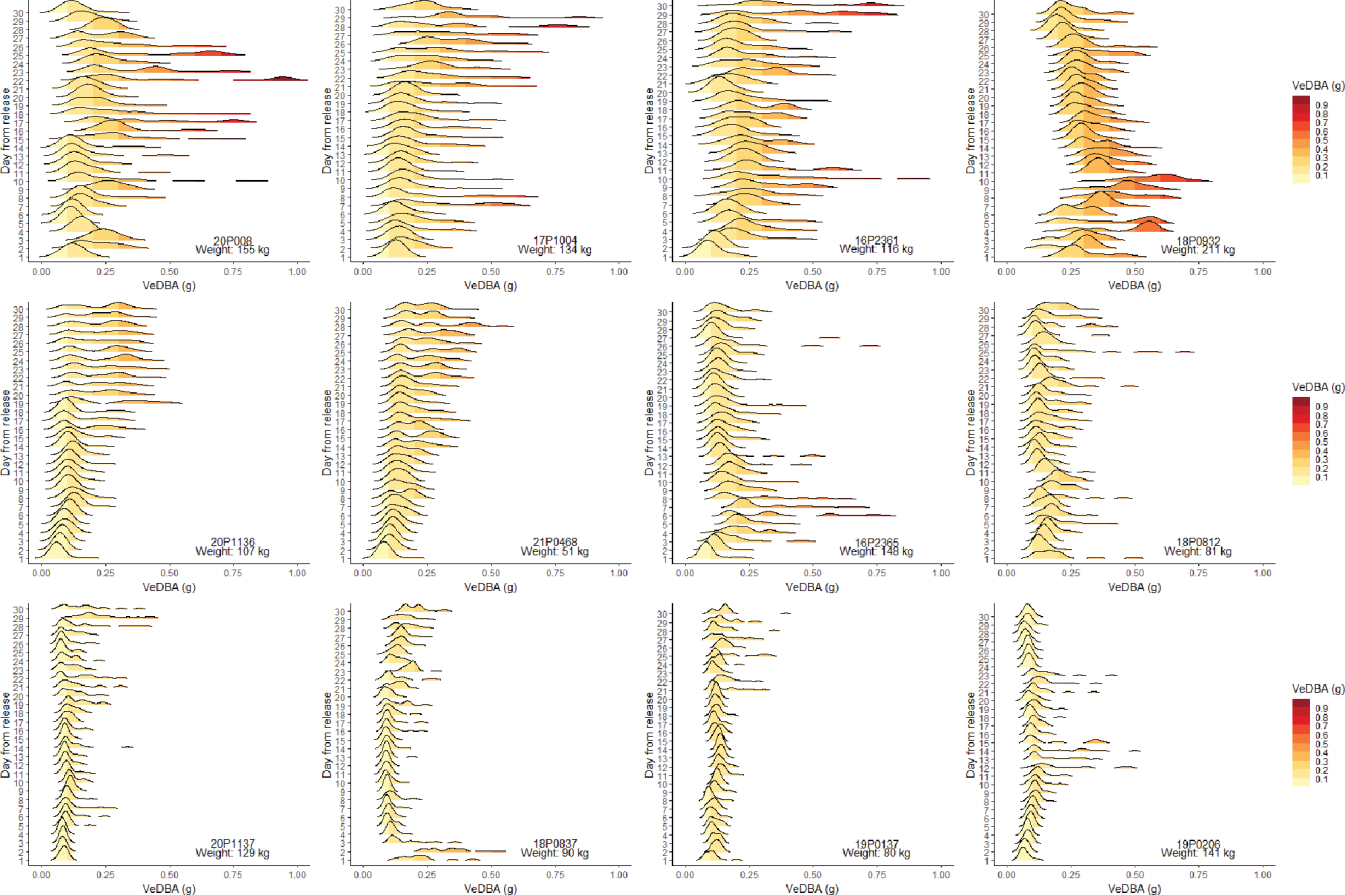
Density distribution of activity by ABT for the first 30 days of deployment. Ridgeline plots of the density distribution of hourly mean VeDBA for the first 30 days following release for all 12 MiniPAT tagged fish. ABT all began with small VeDBA distribution in the first day following release, which increased over time, except for 18P0837, which expended the highest activity in the first two days post-release

**Fig. 8:**
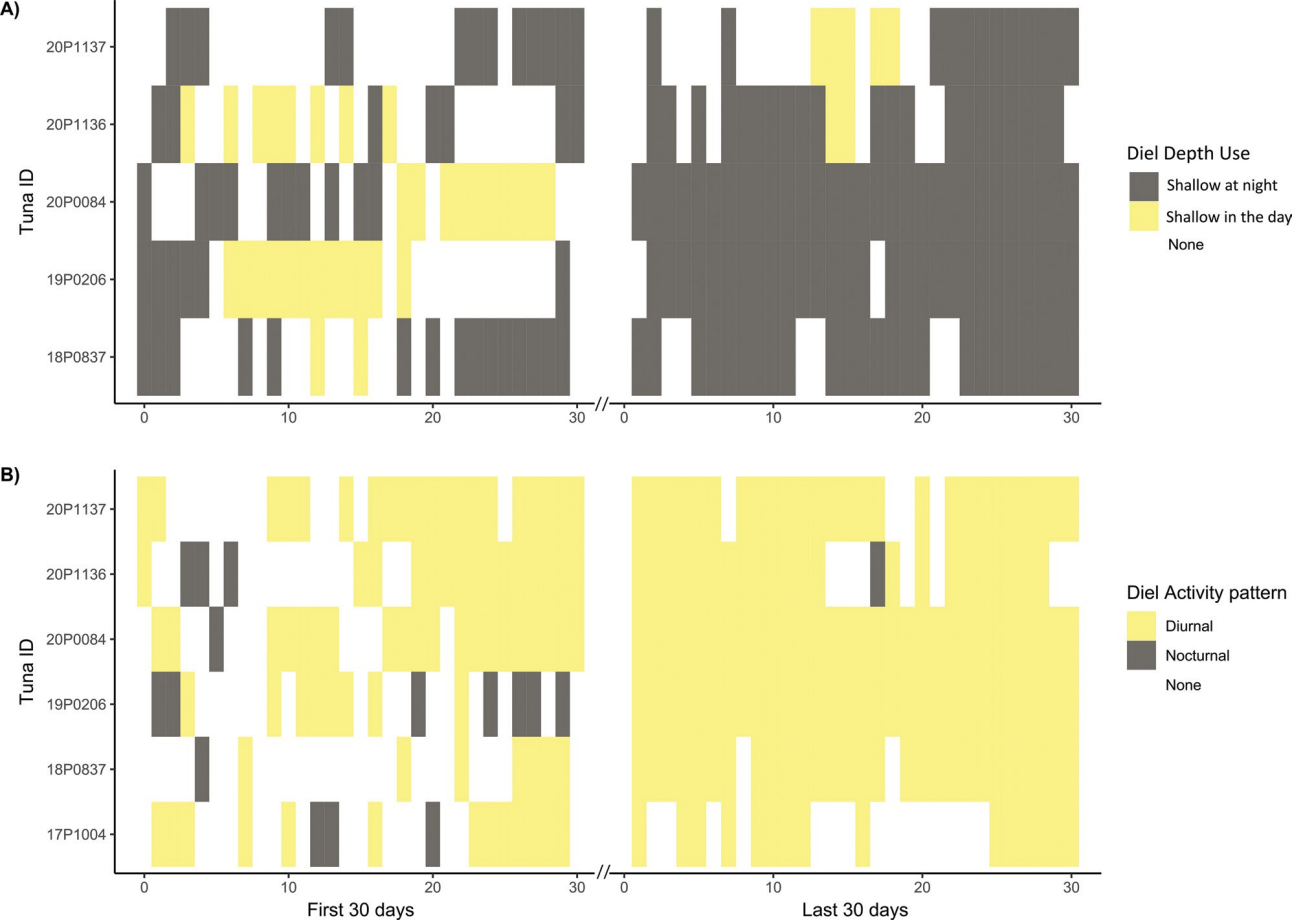
Diel patterns of depth and activity for the first and last 30 days post-release. Plots showing diel patterns in (**A**) depth use, and (**B**) activity pattern, for six ABT tracked for a year, for the first (left column) and last (right column) 30 days of the deployment. ABT 17P1004 was excluded from the depth analysis for the last 30 days of the deployment as the depth sensor became faulty before the end of the tracking duration. Colours represent significant differences in behaviour during night and day (see legend, right), and white colouration where there was no significant difference

The initial burst activity recorded by G7 and MiniPAT tagged fish immediately post-release was followed by a period of very low activity (lower than 95% of all VeDBA data) compared to that observed over the annual cycle. Low activity occurred from 8 h post-release (max onset 21 h), with the lowest mean hourly VeDBA value recorded across the entire 12-month deployment period (for MiniPAT fish; Fig. 6B). In the first 24 h of deployment, mean hourly VeDBA values fell within the lowest 5th percentile of all mean hourly VeDBA recorded across ABT’s full deployments, for an average of 13 ± 10 h, with reduced and dissimilar patterns activity lasting from 2 to 26 days (mean: 11 ± 7.9 days, Figs. 7 and 8). Seven out of 12 ABT (18P0812, 18P0837, 19P0137, 19P0206, 20P0084, 20P1136, 20P1137) exhibited irregular activity for more than a week post-tagging, with no evidence of diel patterns of behaviour (Figs. 2 and 8; Table 2), while three (16P2365, 17P1004, 18P0932) exhibited consistent diel activity within four days (Table 2, Supplementary Figs. 4 & 5). Across all MiniPAT tagged ABT, low daily similarity value ranks were recorded in the initial days following release, with the first two days post-release being the most dissimilar days in four of the 12 fish (Table 2). Following this period of reduced activity, fish increased activity at different rates (Figs. 2, 7, Supplementary Fig. 5).

There was no relationship between fight time or fish length or weight on when diurnal activity resumed (fight time linear regression: R^2^ = 0.03, *p* = 0.61; fish length R^2^ = 0.21 *p* = 0.13; weight: R^2^ = 0.22 *p* = 0.13). Water temperature at capture also didn’t explain the period of time to exhibit diel vertical migration (hereafter DVM) (linear regression: R^2^ = 0.51, *p* = 0.49), and though the timing of DVM patterns coincided with the new moon for six of the 12 ABT, this may be an artefact of when tagging occurred, as seven out of 12 fish were tagged during the waning moon, and an additional four fish during the new moon. Mean activity remained similar regardless of moon phase in the first 30 days post-release (Kruskal-Wallis rank sum test, X^2^ = 0.66, df = 3, *p* = 0.88).

### 30-day behavioural response– depth use

In contrast to reduced activity patterns, only four MiniPAT tagged ABT showed dissimilar patterns of depth use following release (Table 3; Fig. 3). The return of diel depth use in these individuals coincided with a change in their location (and thus the underlying mean bathymetry from 104 ± 15 m to 2,669 ± 948 m), and occurred during different lunar phases. For eight fish, depth use remained similar regardless of lunar phase (Kruskal-Wallis rank sum test, X^2^ = 3.93, df = 3, *p* = 0.27) and was generally better described by the underlying bathymetry, constraining dives (Fig. 3). For example, ABT that experienced a narrow depth range and little variation in mean bathymetry throughout the first month of deployment maintained similar patterns of depth use throughout the first 30 days (Fig. 3, fish IDs: 18P0837, 19P0137, 20P1136, 20P1136, 21P0468). Overall, across the first 30 days, ABT swam shallower at night than during the day (GLMM: t_2_= -2.49, *p* = 0.01; day: 24.5 ± 34.6 m, night: 20.6 ± 17.6 m). However, depth use remained similar regardless of whether ABT were undergoing directed or localised movements as determined by the HMM (GLMM: t_2_=-1.63, *p* = 0.10; directed movement: 22.9 ± 29.5 m, localised movement: 21.5 ± 20.2 m), and there was no interaction between time of day and movement state (GLMM: t_2_=-1.67, *p* = 0.10).

### Measuring fast starts (potential foraging events) and behavioural influences of tagging

ABT varied in the amount of time they allocated per day to fast starts. Inactive days, defined as those when fish spent < 1% of their time performing fast starts, occurred on average for 6.2 ± 1.3 days of the first week post-release (88% of the week, range 4–7 days; Fig. 4B). By week 4, inactive days occurred on 4.3 ± 2.5 days of each week (61% of the week). Overall, in the first 30 days, fish spent 0.76 ± 1.54% of each day performing fast starts. This varied between fish (e.g. fish 20P0084 allocated 14.9%, while fish 20P1137 allocated 0.3%; Fig. 4A). In contrast, in the last 30 days of deployment ABT were much more active, allocating < 1% of each day to fast starts for only 2.7 ± 2.1 days of weeks 1 and 4 respectively (Fig. 4E-F, Supplementary Figs. 6 & 7).

### Behaviour 11 months later

For tags deployed for 12 months (*n* = 6), the last 30 days of the deployment showed diurnal patterns of behaviour (Fig. 8B), with greater VeDBA during the day compared to the night (Wilcoxon rank sum test, W = 3.5 × 10^6, *P* < 0.001, VeDBA_day_: 0.30 g ± 0.14 vs. VeDBA_night_: 0.18 g ± 0.10, Fig. 6C). They were also more active overall during the last 30 days of deployment (VeDBA_dayF_:0.19 g ± 0.13 vs. VeDBA_dayL_:0.30 g ± 0.14), and their mean hourly VeDBA remained consistently higher than the value that was recorded immediately post-release (red horizontal line Fig. 6B and D). Although variable, higher daily similarity values were recorded for all six fish during the last 30 days of deployment (Table 2; Fig. 5S-X). For example, fish 18P0937 and 19P0206 had daily similarity values that were 7 and 17 times greater, respectively, than for first 30 days (18P0937: 0.57 ± 0.09 vs. 0.08 ± 0.13; 19P0206: 0.68 ± 0.08 vs. 0.04 ± 0.16). Depth use was also far more consistent between days and among individuals during the last 30 days (Table 3, Supplementary Fig. 8), and across lunar phases (Kruskal-Wallis rank sum test, X2 = 1.02 df = 3, *p* = 0.80). In contrast to the first 30 days of deployment, mean bathymetry remained shallow throughout the last 30 days of the tracking (mean: 114 ± 80 m; Supplementary Fig. 6) and ABT typically swam significantly shallower at night than during the day.

In terms of spatial behaviour, there were no significant differences in the distance covered by the ABT during the first and last 30 days of deployment (mean_first30_: 1,033 km ± 243 km, range 655-1,477 km; vs. mean_last30_ 845 ± 396 km, range: 460–1452 km, Welch t-test, t = 1.07, *p* = 0.32). Similarly, no significant difference was observed in the overall straightness index (SI_first30_ 0.39 ± 0.20; vs. SI_last30_ 0.24 ± 0.12, Welch t-test, t = 1.90, *p* = 0.08). However, ABT displayed an initial flight response in the first 14 days following release (see results section “Behaviour immediately following tagging”).

## Discussion

Atlantic bluefin tuna appear to exhibit a phased response to capture, tag and release, with a strong, highly active initial swimming response over 5 to 9 h, as reported in other short-term, post-release studies of large fish [15, 44, 45, 57, 65, 66]. This initial response is followed, however, by a period of significantly reduced activity, lacking diel behaviour patterns, lasting from 2 to 26 days (mean 11 ± 7.9 days) before consistent behaviour and activity were re-established. This has not been shown before and has importance both for the treatment of biologging data, and the welfare of large fish during capture and tagging.

### Behaviour immediately following tagging

In the present study, ABT activity, tailbeat amplitude and frequency were 2.4, 3.2 and 1.4 times greater respectively in the first hour post-release than the subsequent 24-hours (Fig. 2). This is comparable to previous findings in ABT, where tailbeat frequencies were higher and sustained in the first hour post-release, before plateauing within 5–10 h [44, 45]. In the current study, mean hourly VeDBA values were on average 1.3 times greater for MiniPAT compared to G7-tagged ABT, which we suggest is likely due to the tag anchoring system. G7 tags were anchored front and aft to reduce tag wobble, resulting in lower standard deviations for mean hourly VeDBA compared to the MiniPAT tags, which were anchored front and centre. This difference in anchoring may have resulted in the MiniPAT moving more on the fish, and contributing to the greater variability and differences in hourly mean VeDBA (Fig. 6A).

The process of capture for large fish is significant, as fish are often fought to the point that they can no longer overcome the resistance of the fishing gear. Burst activity during the fight is most likely powered by anaerobic metabolism, accumulating metabolic end-products such as lactate and cortisol, and decreasing blood pH [11, 67–70]. During this period, fish can incur hooking injuries [41, 71, 72], and capture may also elicit other physiological stress responses [69, 73]. The effects of the tagging process, from air exposure during boarding the fish, to biological sampling and tag incision, will also have an impact on the ABT [69, 74, 75]. Given these impacts, fight times and short handling durations (mean 2.6 ± 0.5 min) were minimised in the current study. ABT were irrigated whilst on deck, and ventilation was assisted through reoxygenation tows following assessment for fitness for tagging and release. It is important to point out that while there can be sub-lethal impacts of tagging on ABT, this study recorded zero mortalities, consistent with post-release mortalities reported for ABT [58] and Pacific bluefin tuna (*Thunnus orientalis*, hereafter PBT) [3, 37, 41–43, 46, 70, 76].

Based on physiological studies of fish, the post-release recovery phase encompasses restoration of homeostasis, including replenishment of oxygen and glycogen stores, and removal of excess blood lactate [67, 77] which may take long periods of time [69]. For example, fish cortisol levels are generally thought to peak within 1–2 h following intense activity, and the recovery of muscle lactate and glycogen can take up to 12 h, depending on the species [67, 78, 79]. This might be aided by swimming faster for some portion of the recovery period, as shown in blue marlin (*Makaira nigricans*) and sailfish (*Istiophorus platypterus*) [55, 57], ten ram-ventilating shark species and ABT [16], and by the initial prolonged dive and rapid dominant stroke frequencies (Fig. 2C) made by tuna in the present study. These dives may be associated with increasing oxygen intake but perhaps might also aid in thermoregulation. Blue marlin, for example, may gain up to 2.1° C following a 15-minute fight on rod and reel [55]. Thus, ABT in the present study may have also been diving to cool down following capture. Muscle temperatures as high as 29.4 °C have been reported in small PBT following capture, which dropped by 5 °C within 40 min of release [80]. While speed was not measured in the present study, a relationship between tailbeat frequency and speed has previously been directly recorded in ABT carrying biologging tags that included speedometers, accelerometers and depth sensors [45]. The direct energetic cost of swimming at faster speeds following release has been estimated to make up to 47% (± 9%) of ABTs daily energy expenditure [16].

### Behaviour post 24-hours

It is generally accepted that the first 5–10 h post-release are influenced by a stress response to capture, handling and/or tagging [16, 45, 81, 82], but biologging data following this are generally considered to represent “normal” behaviour. Instead, we show that ABT exhibit a subdued activity state with loss of diel behaviour, lasting between 2 and 26 days (on average 11 days) (Figs. 3B and 5), with the duration and the magnitude of this response varying between individuals (Fig. 6). This includes the proportion of time allocated to fast starts (Fig. 4A-C), which may represent a variety of behaviours in ABT, including feeding. This reduction in overall activity may be because the ABT spent more time than normal making directed migration movements in the first two weeks post-release, and therefore did not exhibit the usual activity associated with foraging. Further work using internal archival tags to monitor foraging and acceleration simultaneously, as has been done in captive ABT (see [60]) would help clarify this. Internal tagging can directly measure the heat increment of digestion [61, 83, 84] and has previously been used to suggest that ABT and PBT can resume foraging within a few days to a week post-tagging [36, 60, 85, 86]. For example, southern bluefin tuna (*Thunnus maccoyii*) resumed feeding on average 19 days after release (range 5–38 days; [87]). The subdued activity state may also be a response to tagging itself, with behavioural changes associated with exposure to stressors such as handling and air exposure impacting the preference or ability to feed [88]. ABT may have taken time to become accustomed to the tag attachment and diverted metabolic pathways to wound healing [89]. Short-term impairments in swimming performances, such as decreased critical swim speeds (the maximum speed a fish can maintain, representing the upper limit of aerobic swimming performance), slower tailbeat frequencies and reduced aerobic scope, have been observed in several species with external tags compared to their non-tagged counterparts [90, 91]. These effects were attributed to increased drag or the tagging procedures [92–94]. Other studies have found no significant differences in swimming performance between tagged and untagged fish [95–97], however, measurements were conducted 24 h to 7 days post-tagging, by which point the initial response to tagging may have subsided.

### Behaviour 11 months later

In contrast to the rapid and direct movement following tagging (Fig. 4A), a year later, four ABT were resident within the English Channel, one was migrating back from the Bay of Biscay to the Channel, and one migrated away from the Channel to the west coast of Ireland, but at a much slower rate than movements recorded in the first week post-tagging (58 km.day^− 1^ vs. 73 km.day^− 1^) (Fig. 4D). The greatest distance travelled always occurred within the first 24 h of the deployment, and these initial movements may have been to escape the capture and tagging location (a flight response [88]), and perhaps a period of increased ventilation to facilitate the removal of metabolic end-products. Black marlin (*Istiompax indica*) tagged in the Coral Sea were found to exhibit similar rapid movement up to 556 km away from their release sites [98]. Sharks (eight different species) also appeared to swim offshore to deeper water following capture, although displacement distances varied between species [99]. However, in other ABT and PBT studies, shorter displacement distances were recorded after release [42, 70, 100, 101] with fish remaining in the vicinity of the release locations [102], particularly in smaller individuals [103]. In the present study, directed movements appear to be either too early in the year to be characterised as part of the general migratory cycle or too large to be characterised as day-to-day dispersal between feeding areas while resident in the English Channel based on data from individuals tracked from the English Channel for over a year (481–708 days) [58]. However, further work is required to define the extent and nature of any post-tagging escape response.

Almost a year after capture, ABT in the present study returned to the same waters and exhibited relatively consistent diel vertical migration in shallow (< 200 m) waters (Fig. 4D, Supplementary Fig. 8). In contrast, in the first 30 days post-release, five of the 12 ABT remained in shelf waters but did not show any diel diving behaviour (Fig. 5, Supplementary Fig. 4). Similarly, ABT tracked from the western Atlantic also showed no diel vertical migration following release [36, 104, 105] perhaps, as in the present study, because they were released over shallow shelf regions (~ 20 m depth to the bottom [36, 100, 104]). Depth use remained similar across the lunar cycle (Fig. 3), which was perhaps surprising as the lunar cycle’s influence on depth use has been observed in PBT [106, 107], Southern bluefin tuna [87] as well as ABT [100, 101]. Instead, differences in the period of behavioural disruption may be attributed to factors such as maturity, time since feeding, and intrinsic physiological differences between individuals [15, 108].

Thus, each capture event has its own conditions that begin with how the fish are captured, how long they are handled for, and how they are tagged, which may all impact recovery differently. The duration of the post-release response may be proportional to the magnitude of the stressors [67] and linked to fish size, with larger fish exhibiting more prolonged responses to capture and tagging [70]. This may be due to larger fish taking generally longer to land, increasing the relative cost of the fight [67]. However, this did not appear to be the case in the present study, nor in blue marlin, sailfish, and greater amberjack (*Seriola dumerili*) [19, 57]. The intensity of the fight may be a more important factor than fight duration, although making an objective measure of ‘fight intensity’ is extremely challenging. Future work may include fishing gear fitted with accelerometers to measure the intensity of capture events [13, 109]. ABT in the current study may have recovered more quickly from the physical exercise associated with capture than the time required to adjust to tagging. Electronically tagged ABT have high survival rates [8, 41, 46] are known to resume feeding within days following tagging [60, 86, 110] and perform important life history events such as spawning whilst tagged [39, 47, 48].

### Challenges in defining recovery

A key challenge in biologging is understanding when the host animal is likely to exhibit behaviour that is representative of the broader, untagged population. Removing the ‘tagging artefact’ from data is essential, yet metrics used to define the duration of the post-release period are highly variable [15, 111–113], ranging from physiological markers [69, 70], metabolic activity [114] and behaviour [19, 56, 57], which may all be influenced by the duration and resolution of measurement [30, 79]. While approaches such as recapturing tagged individuals [115, 116] can help to isolate the effect of capture and tagging, this method may not be feasible for highly mobile species where recapture rates are low. In the present study, we found that return to baseline behaviour was variable between individuals, with post-release behaviour being altered for several days, and in some cases weeks, rather than hours. In several bluefin tuna studies, the first 1–6 days have been discarded to allow for the fact that feeding or behaviour may be altered immediately after capture and tagging [61, 84, 106], though several studies with implanted archival tags revealed rapid return to feeding within days of tagging [60, 83, 86] highlighting the differences in response to capture and tagging even within species. In the present study, these patterns were influenced not only by time since release, but also by factors such as fight time, fish size and possible tag burden. Here we suggest that short-term deployments (i.e. less than a week) may fail to capture unaffected behaviour in some cases [19]. Our results highlight the value of combining long-term deployments in tandem with high-resolution tags to identify and quantify short-term effects that may otherwise be missed.

### Impacts of capture and tagging

The results here suggest that ABT may have a more complex response to capture, tagging and release than previously considered [17, 117]. Minimising interaction [69, 70, 102] is probably sensible, as ABT increased the duration of their initial recovery dive by 4 min with each additional minute fighting the line, though fight time did not predict when the fish would resume diurnal behaviour. This may be due to issues beyond the present study, such as inflammation, discomfort and what fish may experience as pain from the intra-muscular tag darts or biological sampling (fin clip and muscle biopsy). Local tissue damage associated with insertion of internal darts have been reported in different tag attachment methods [74, 75], and wound healing can be delayed when fish are subjected to other stressors [118]. Nociception has been seen to affect behaviour in fish including delay in reception of feeding and loss of equilibrium [119]. Local analgesics can block nociception in fish [120, 121], thus future work could investigate the effect of tagging itself and whether the use of local analgesics may reduce the impacts of tagging in ABT.

## Conclusion

In summary, the impacts of capture, handling and tagging on ABT have probably been generally underestimated owing to limits on the length of many studies, yet we show that the effects of capture may extend from at least days to several weeks before ABT resume “normal” behaviour. The drivers of this remain unclear and complex, with likely interactions between fight times and intensity, handling, as well as response to tagging and wound healing. Additional factors including fishing gear, angler experience, tag attachment time, whether fish were boarded, abiotic conditions (such as temperature, dissolved oxygen and bathymetry) and condition of fish prior to capture may also have an influence. Further long-term studies may reveal if this is also the case in different species, with important implications from ethics to data analysis, and eventually effective stock management.

## Electronic supplementary material

Below is the link to the electronic supplementary material.

Supplementary Material 1

## Data Availability

Tracking data will be made available upon acceptance of the manuscript on Movebank and the Cefas Data Portal.
