# Supplementary material for: Use of accelerometry to measure the dynamics of activity patterns of Atlantic bluefin tuna after tagging and release
Source: Mov Ecol. 2025 Jun 5;13:37. doi: 10.1186/s40462-025-00563-4 (PMC12139206; doi:10.1186/s40462-025-00563-4)
Supplement: Supplementary file 1 — Supplementary Material 1 [file 40462_2025_563_MOESM1_ESM.pdf]

CATEGORY	DESCRIPTION	SCORE	FITNESS TO ENTER	FITNESS FOR RELEASE
ACTIVITY & MOVEMENT	**Alert** *e.g.* body twitching, finlet movement, frenetic movements, muscle rigour and tone, responds to visual stimulus.	3		
	**Lethargic** *e.g.* animal slow to respond, some muscle tone, but lacking vigour.	2		
	**Moribund** *e.g.* complete lack of vigour and tone, no muscle movement involuntary or provoked, no response to visual stimulus.	1		
PHYSICAL APPEARANCE	**None/Minor** *e.g.* No apparent markings or minor old (fully healed) scars or scraping or rubbing of body from fishing line without perforating skin.	3		
	**Moderate** *e.g.* superficial lacerations from fishing line or other. Main locomotory fins and body parts (inc. jaw, eyes, gill plates etc.) present and function.	2		
	**Major** *e.g.* **s**ignificant injuries or pathology *i.e.* significant evidence of infection or other pathology that limits future health and welfare.	1		
BLEEDING	**None/minor** *e.g.* none or minor blood loss associated with the use of hooks, lip-hook and sampling/ tagging procedures.	3		
	**Moderate** *e.g.* continuous seeping of blood from hidden or visible wounds, but no pulsating bleeding indicative of arterial puncture. Blood from sampling sites heavily stains water on deck.	2		
	**Severe** *e.g.* venous/arterial puncture, pulsating bleeding. Blood forms dense viscous pools on deck or significantly colours sea water.	1		
DURATION ON DECK (mm:ss)		SUM OF SCORES		
**NOTES:**		**HOOKING LOCATION**		

Supplementary Figure 1: Visual health assessment form used to score the condition of the fish prior to and after tagging. Fish may only enter the study if the "fitness to enter" score is above 6 and can only be released as part of the study if the "fitness to release" score equals the "fitness to enter" score.

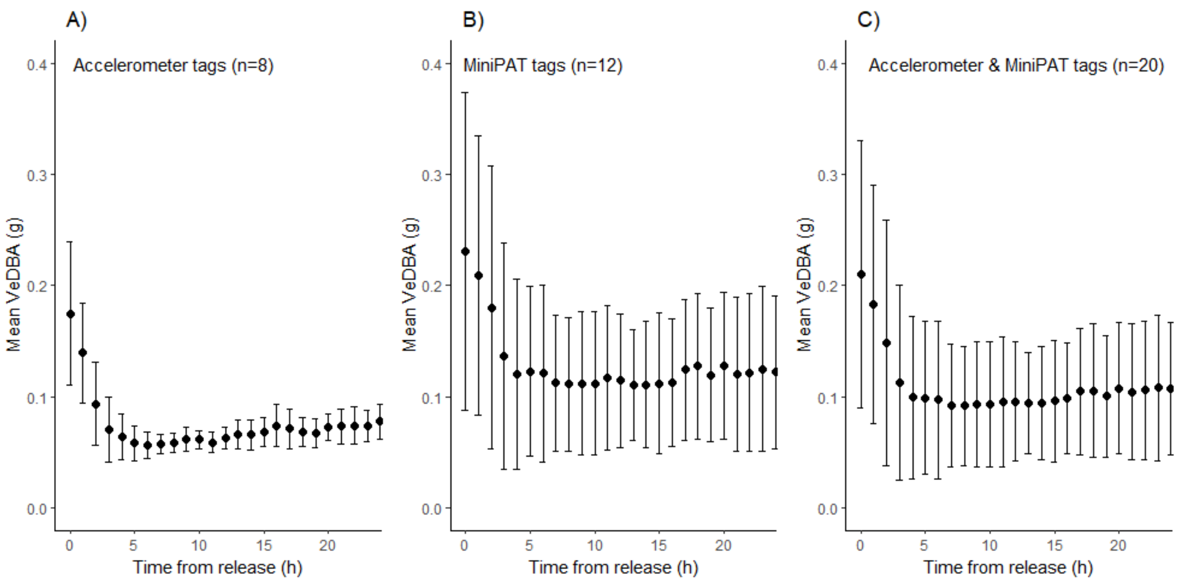

Supplementary Figure 2: **Mean hourly VeDBA by tag type**. Mean hourly VeDBA for the first 24 hours following release for A) the G7 tagged fish (n=8), B) the MiniPAT tagged fish (n=12), and C) for all fish combined (n=20). G7 tags were down-sampled to 0.2 Hz to match the resolution of the MiniPAT tags. Vertical bars represent the standard deviation. Higher variation observed in the MiniPAT tagged fish is likely to result from the difference in tag anchoring.

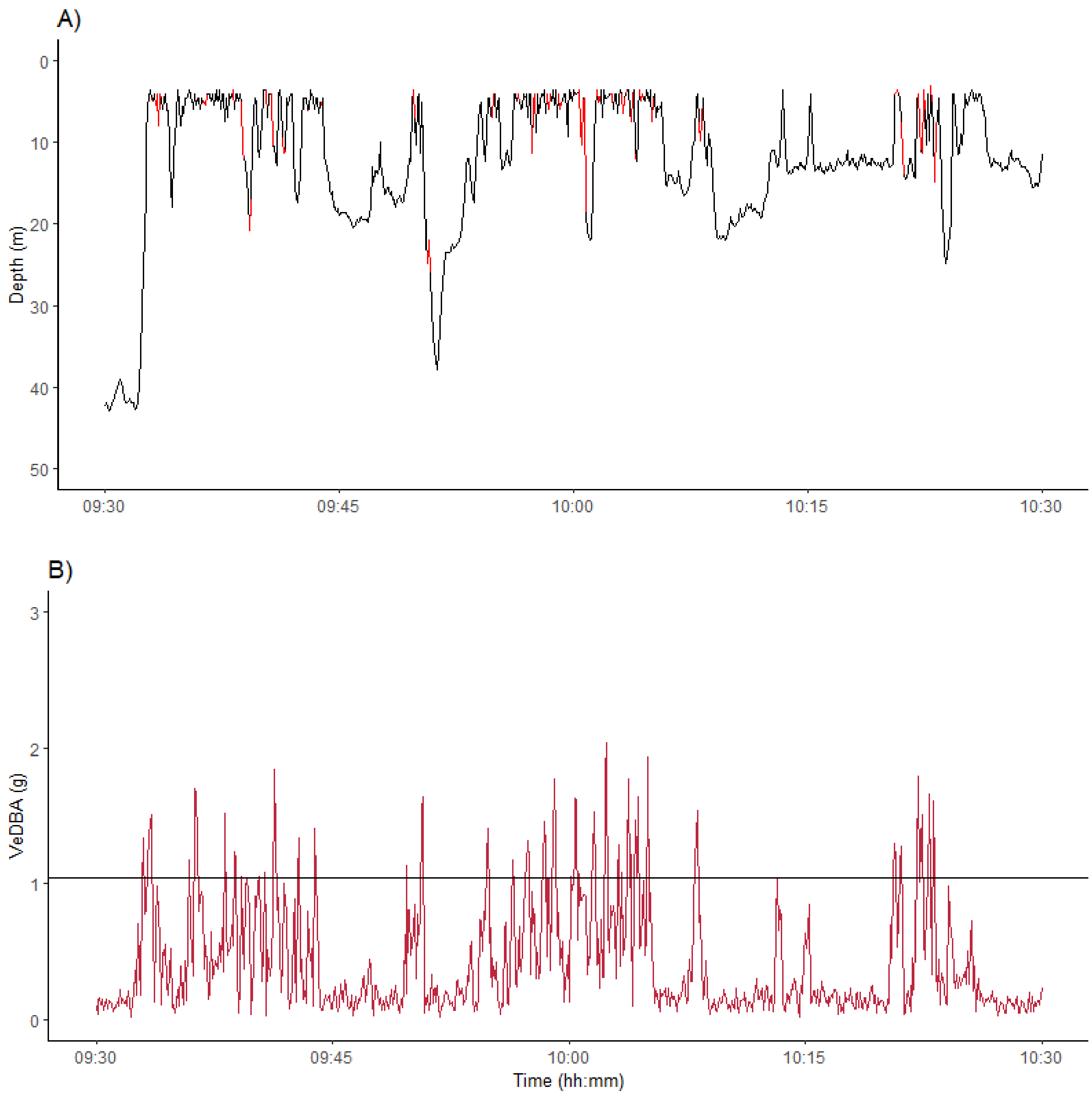

Supplementary Figure 3: Fast start event characterisation. Example of fast start events in function of depth and activity levels. (A) Depth profile of an ABT where fast start events (i.e. periods of when VeDBA > 99th percentile) are coloured in red. (B) the associated time series of activity where the horizontal line corresponds to the threshold above which activity is considered a fast start.

A)

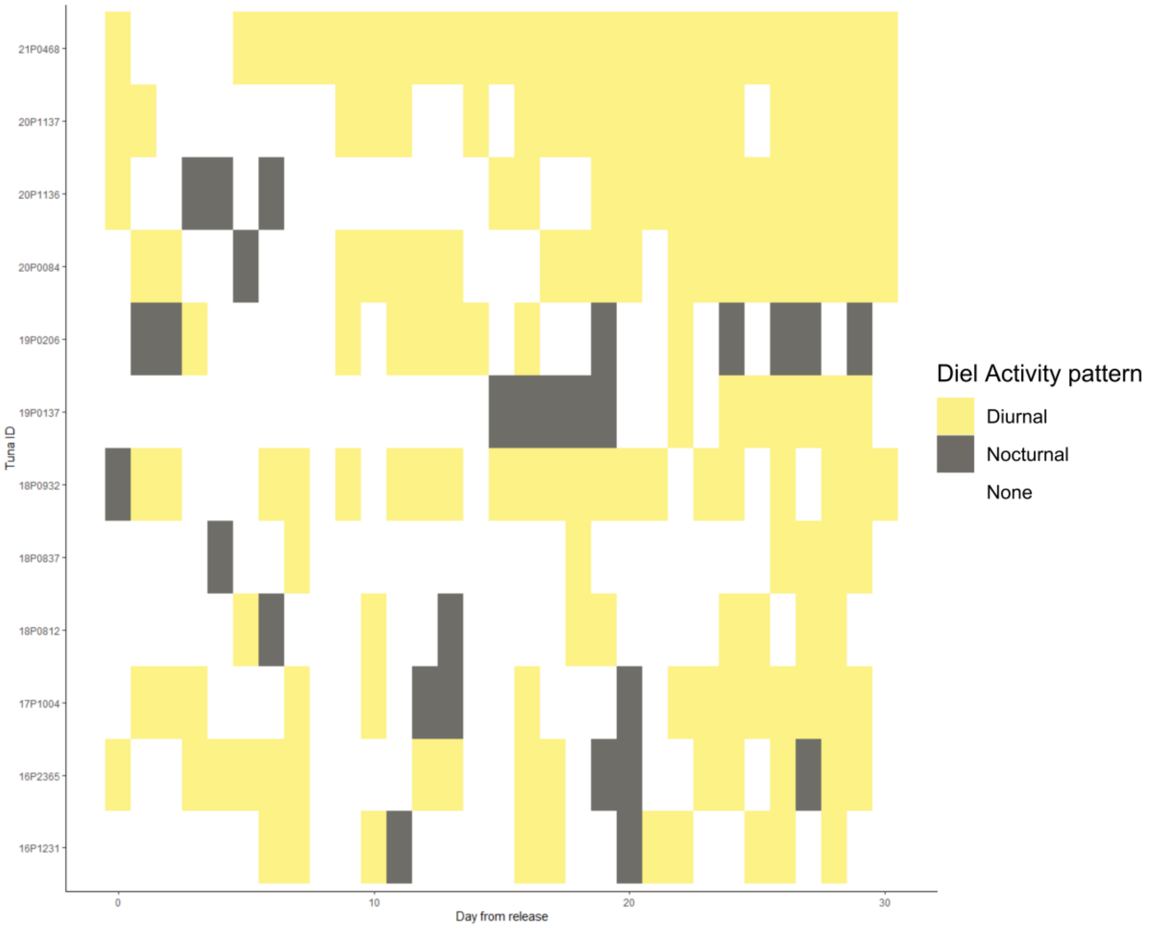

21P0468
20P1137
20P1136
20P0084
19P0206
19P0137
18P0932
18P0837
18P0812
17P1004
16P2365
16P1231
Tuna ID
0
10
20
30
Day from release
Diel Activity pattern
Diurnal
Nocturnal
None

B)

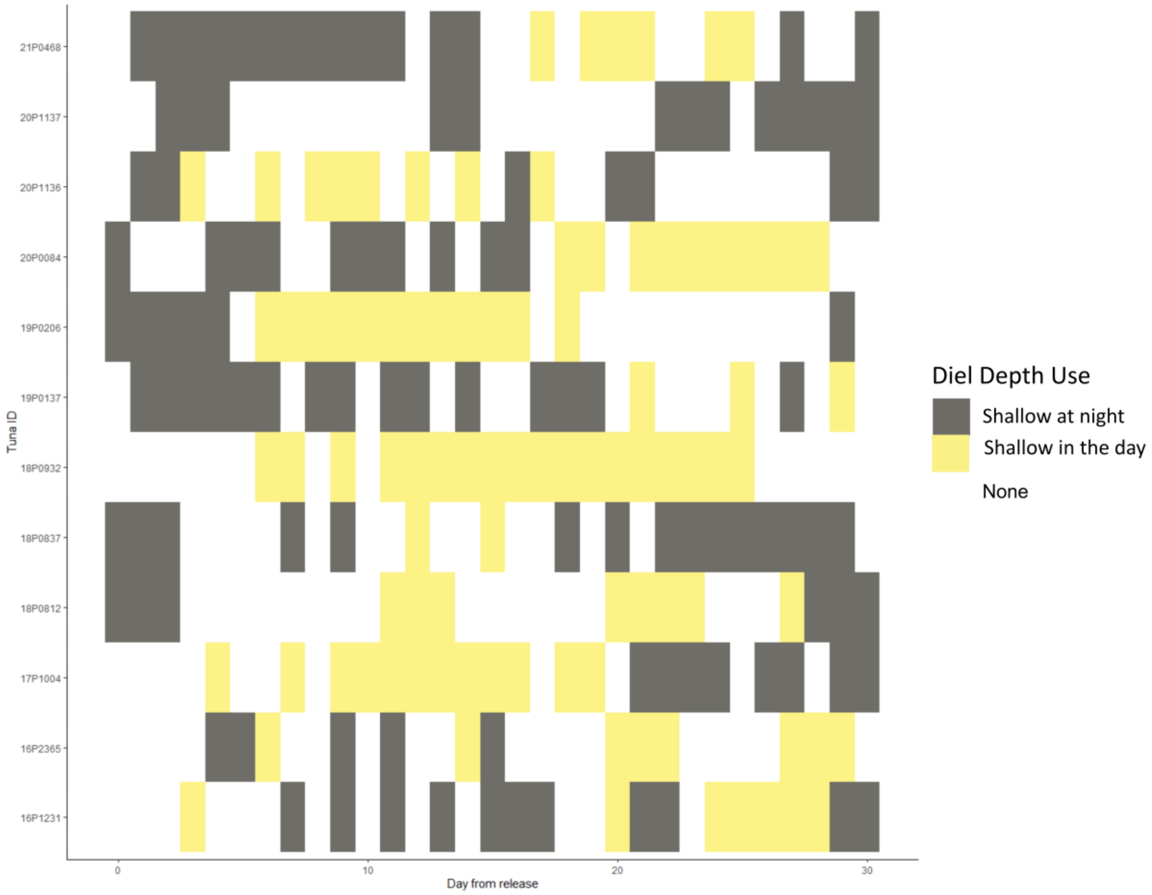

21P0468
20P1137
20P1136
20P0084
19P0206
19P0137
18P0932
18P0837
18P0812
17P1004
16P2365
16P1231
Tuna ID
0
10
20
30
Day from release
Diel Depth Use
Shallow at night
Shallow in the day
None

Supplementary Figure 4: **Diel patterns of depth and activity for 12 ABT for the first 30 days post-release.** Plots of the diel patterns of activity (A) and of depth (B) by ABT tagged with MiniPAT tags (n=12) for the first 30 days following release. Colours represent significant differences in behaviour during night and day, and white colouration where there was no significant difference. For ABT activity (A), yellow boxes represent when fish were significantly more active during the day, while dark grey boxes represent fish being significantly more active at night than during the day. There was little difference in activity between night and day in the first 10 days after which some fish begin to exhibit significant diurnal patterns of activity which became more pronounced after day 16. For diving behaviour (B), dark grey represents diel vertical migration, where ABT swam significantly shallower at night than during the day. Yellow boxes represent days where ABT exhibited reverse diel vertical migration, shallower during the day and deeper at night. In the first 30 days following release, ABT displayed different diving behaviours and did not show any consistent patterns.

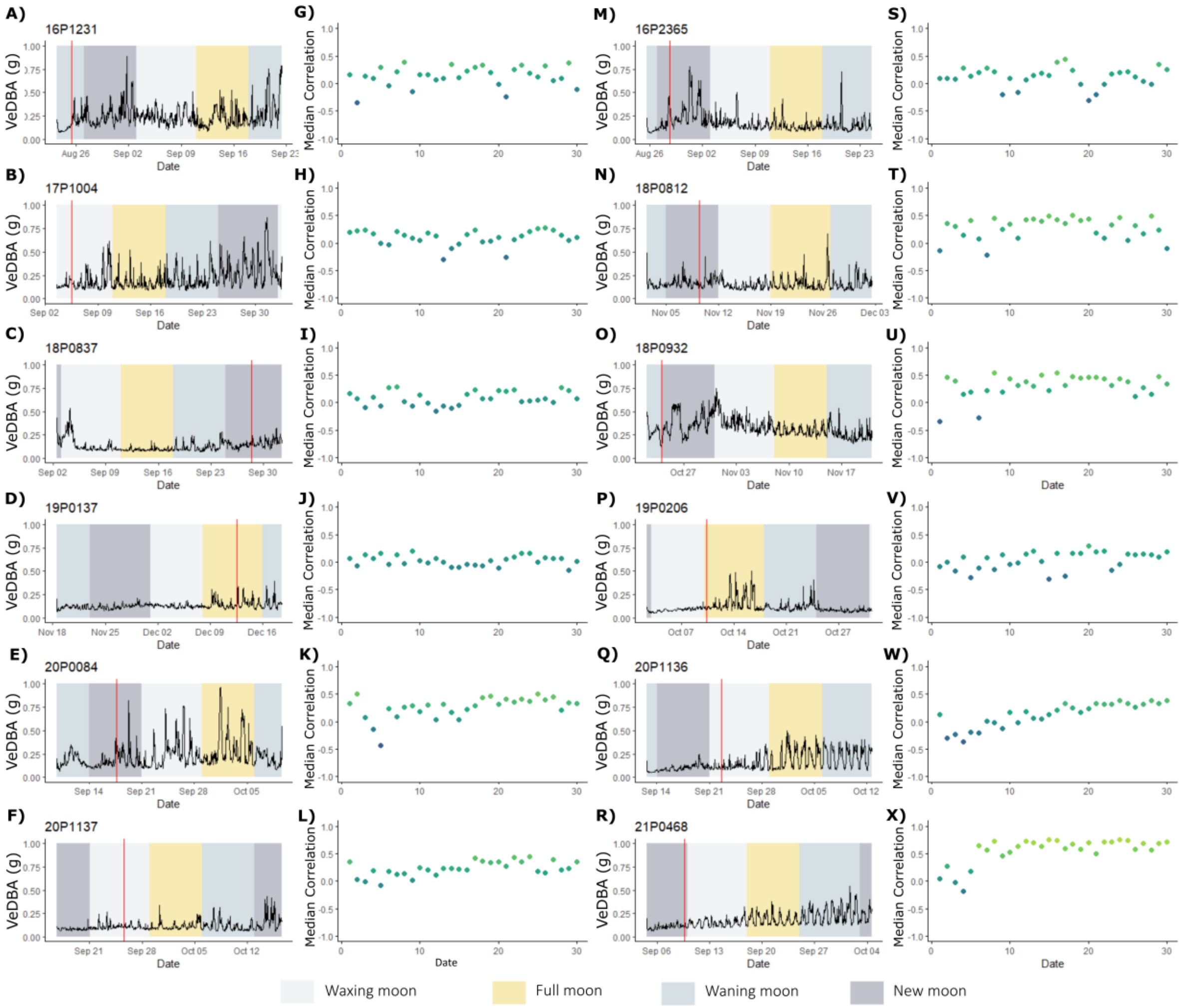

Supplementary Figure 5: Mean hourly activity patterns for 12 ABT for the first 30 days of deployment. Mean hourly activity (VeDBA, first and third columns) of all 12 MiniPAT tagged fish (A-F) and (M-R) for the first 30 days of deployment. Shaded backgrounds corresponded to the lunar phase (see legend, right). Red vertical lines correspond to the time of recovery. Daily similarity values (second and fourth columns, (G-L) and (S-X)) of each day post-release, relative to the whole deployment period (see Methods) for the first 30 days (G-L) and last 30 days (S-X), coloured by correlation values, where days more similar to the overall activity pattern are shown in lime green, and more dissimilar days in blue.

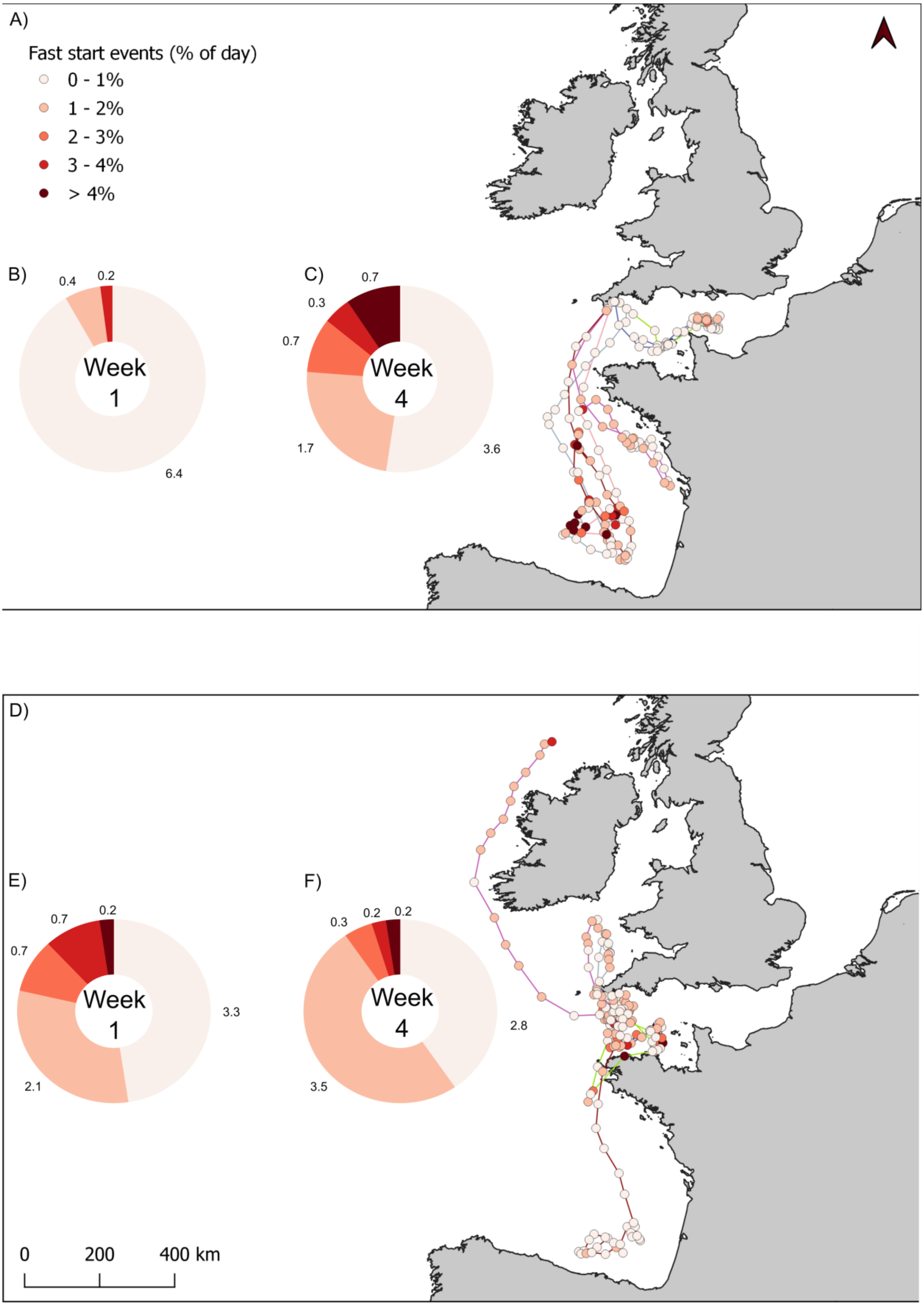

Supplementary Figure 6: **Time allocation to fast start events for the first and last 30 days of deployment.** Maps of ABT daily locations for the 6 ABT with yearlong deployments for (A) the first and

(D) last 30 days of the deployment. Daily locations are coloured by the proportion of each day spent conducting fast start events (the percent of time that VeDBA was above the 99th percentile of values across the full tracking deployment per ABT). Ring plots represent the average number of days per week that ABT allocated to fast starts events, for the first 7 days (B & E) and last 7 days (C & F) of each 30-day period.

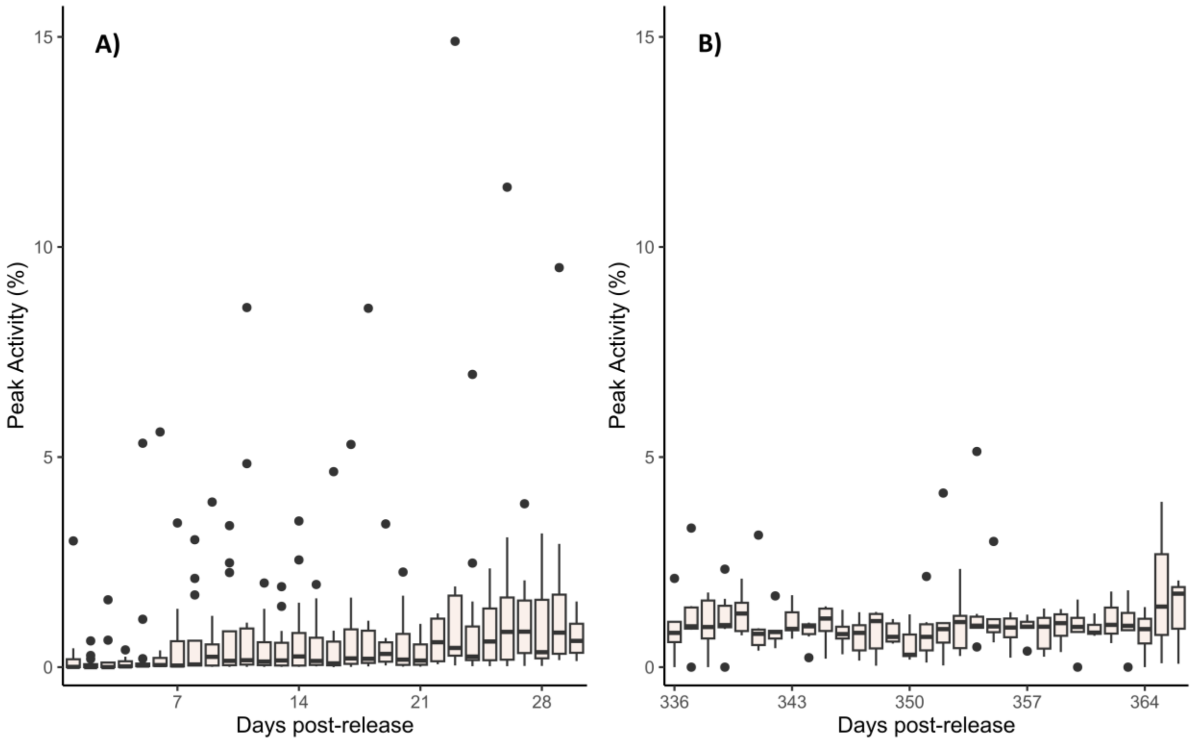

Supplementary Figure 7: Time allocation to fast start events for the first and last 30 days of deployment. Boxplot of the average proportion of time allocated to fast start events by day for (A) the first 30 days post-release (n=12) and (B) the last 30 days post release (n=6). Vertical bars represent the standard error.

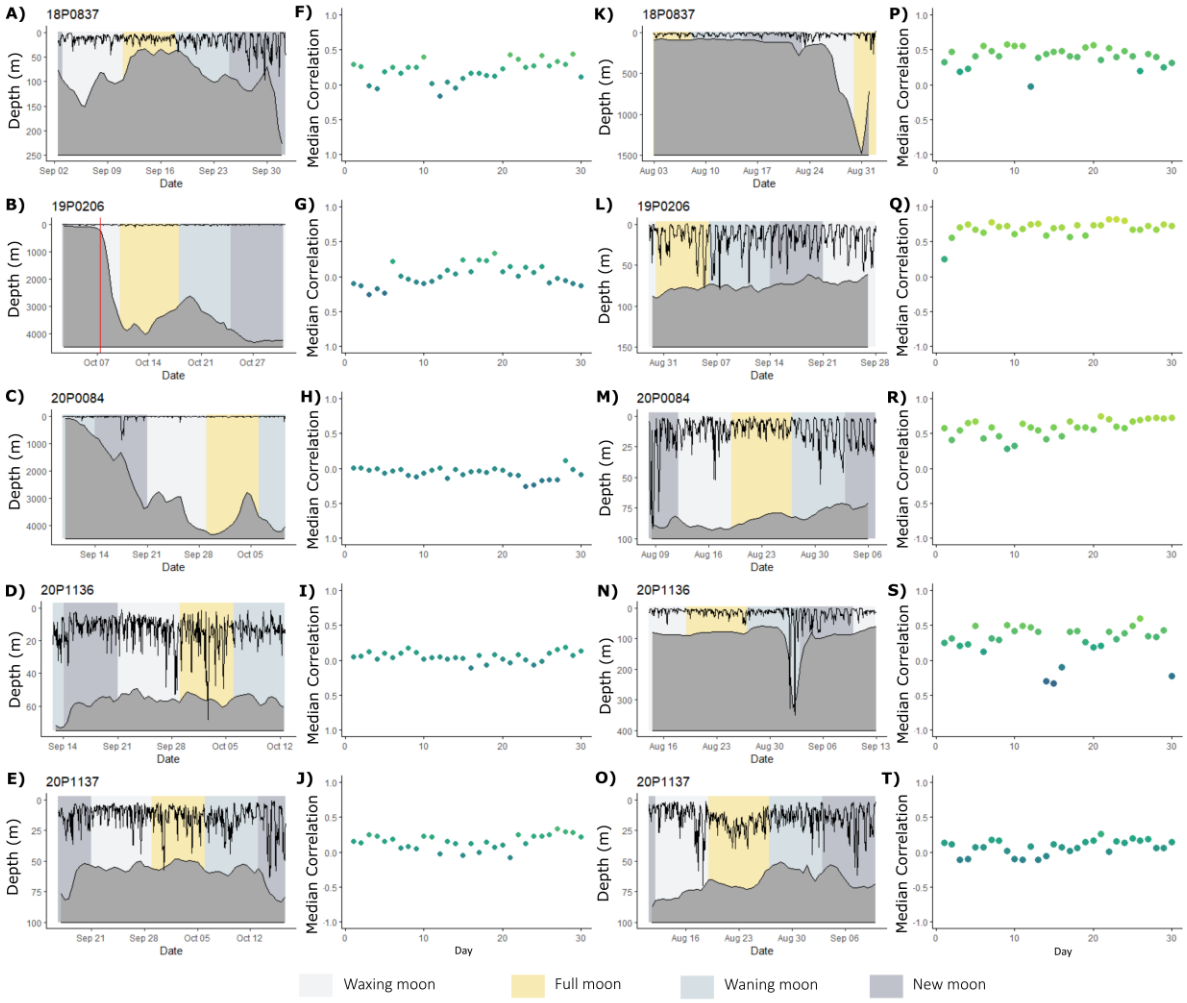

Supplementary Figure 8: **Depth use for the first and last 30 days of deployment for 5 ABT.** Mean hourly depth profile (first and third columns) of five MiniPAT tagged ABT for the first 30 (A-E) and last 30 days of deployment (K-O), with underlying bathymetry shown as a grey polygon. Shaded rectangles correspond to the lunar phases (see legend, right). Red vertical lines (for 4 fish) show the day on which depth use patterns become similar to the overall tracking period. Plots lacking red lines (8 fish) did not exhibit disrupted depth patterns. Associated daily similarity values (second and fourth columns) of each day post-release relative to all other days (see Methods), coloured by their corresponding median correlation values, where days which are more similar are shown in lime green, while more dissimilar days are in dark blue. Fish 16P2365, 17P1004, 18P0932 and 19P0206, exhibited altered activity patterns in the first 7 days post-release, followed by periods of correlated diel patterns of activity. Comparatively, the remaining eight fish did not display consistent altered

behaviour within the first 7 days, with days randomly associated with the overall depth use pattern (values close to 0).
